# Proceedings of the 9th International Symposium on MDS and SAA in Childhood: Athens, Greece. 30 September-2 October 2021

**DOI:** 10.1186/s40348-021-00125-9

**Published:** 2021-10-11

## 2. Do Gata2 dynamics and levels matter during hematopoietic (stem cell, progenitor or other) development?

### C.S. Vink^1^, A. Popravko^1^, C. Eich^1^, A. Maglitto^1^, F.J. Calero-Nieto^2^, W. Jawaid^2^, X. Wang^2^, S.A. Mariani^1^, B. Göttgens^2^, E. Dzierzak^1^

#### ^1^Centre for Inflammation Research, Queen’s Medical Research Institute, The University of Edinburgh, UK; ^2^Department of Haematology, Cambridge Institute for Medical Research, Wellcome Trust-MRC Cambridge Stem Cell Institute, University of Cambridge, UK

Hematopoietic stem cells (HSC) are responsible for life-long maintenance and regeneration of the adult vertebrate blood system. The first HSCs arise from intra-aortic hematopoietic cluster cells (IAHC) in the mouse aorta at embryonic day (E)10.5 through a transdifferentiation process called endothelial-to-hematopoietic-transition (EHT). Gata2 is one of several transcription factors pivotal to the development of the adult hematopoietic system and is required for HSC generation in the embryo. *Gata2*^*-/-*^ mouse embryos suffer lethality at E10.5 and *Gata2*^*+/-*^ HSCs are qualitatively defective. Utilizing our *Gata2Venus* reporter mouse (Kaimakis et al., Blood, 2016) for time-lapse imaging of EHT *in vivo*, we found rapid pulsatile Gata2Venus expression level changes in single transitioning cells, implicating transcriptional instability during establishment of hematopoietic fate (Eich et al., J Exp Med, 2018). Flow cytometric analysis of IAHCs revealed cells with varying levels of Gata2Venus (V^medium^, V^high^) expression. Bulk RNA sequencing of V^med^ showed enrichment of genes involved in leukocyte extravasation, whereas V^hi^ cells upregulated mature myeloid-type genes. Hematopoietic progenitors (HPC) were highly enriched in both fractions, and HSCs were exclusively in the V^med^ fraction. Single cell RNAseq of IAHCs (CD31^+^ckit^+^V^med^) revealed surprising heterogeneity as represented by five distinct transcriptomic clusters (Vink et al., Cell Reports, 2020). Functional HSCs could be refined with SSC, CD27 and Ly6A phenotypic parameters in one major cluster to reveal the transcriptome of the first functional HSCs. Immunostaining of mouse embryos localized these HSCs to aortic clusters containing 1-2 cells. The very low frequency of HSCs in the IAHCs (1/600 amidst the 300/600 frequency of HPCs) highlights the complexity in programming HSC identity as defined by repopulating function, and raises the question – what processes influence acquisition of this rare HSC identity rather than the more abundant closely-related HPC or other identities? I will discuss these issues and the results of single cell methodologies that suggest a stochastic process of quantitative molecular states leading to the establishment of HSC fate/function.

## 4. Hematopoietic Stem Cell Transplantation in Children and Adolescents with GATA2-Related Myelodysplastic Syndrome

### R. Bortnick^1,23^, M. Wlodarski^1,2^, V. de Haas^3^, B. De Moerloose^4^, M. Dworzak^5^, H. Hasle^6^, R. Masetti^7^, J. Starý^8^, D. Turkiewicz^9^, M. Ussowicz^10^, E. Kozyra^1^, M. Albert^11^, P. Bader^12^, V. Bordon^4^, G. Cario^13^, R. Beier^14^, J. Schulte^15^, D. Bresters^16^, I. Müller^17^, H. Pichler^5^, P Sedlacek^8^, M. G Sauer^18^, Marco Zecca^19^, G. Göhring^20^, .A. Yoshimi^1^, P. Noellke^1^, M. Erlacher^1,21^, F. Locatelli^22^, C.M.Niemeyer^1,21^, B. Strahm^1^ for EWOG-MDS

#### ^1^Department of Pediatrics and Adolescent Medicine, Division of Pediatric Hematology and Oncology, Medical Center, Faculty of Medicine, University of Freiburg, Freiburg, Germany; ^2^Department of Hematology, St. Jude Children’s Research Hospital, Memphis, TN, USA; ^3^Princess Maxima Center, Diagnostic Laboratory / DCOG Laboratory, Utrecht, The Netherlands; ^4^Department of Pediatric Hematology-Oncology and Stem Cell Transplantation, Ghent University Hospital, Ghent, Belgium; ^5^Department of Pediatrics, St. Anna Children’s Hospital, Medical University of Vienna, Vienna, Austria; ^6^Department of Pediatrics, Aarhus University Hospital Skejby, Aarhus, Denmark; ^7^Department of Pediatric Oncology and Hematology, University of Bologna, Bologna, Italy; ^8^Department of Pediatric Hematology and Oncology, Charles University and University Hospital Motol, Prague, Czech Republic; ^9^Department of Pediatric Oncology/Hematology, Skåne University Hospital, Lund, Sweden; ^10^Department of Bone Marrow Transplantation, Oncology and Hematology, Wroclaw Medical University, Wroclaw, Poland ;^11^Department of Pediatrics, Dr. von Hauner Children’s Hospital, University Hospital, LMU, Munich, Germany; ^12^Department for Children and Adolescents, Division for Stem Cell Transplantation and Immunology, University Hospital Frankfurt, Frankfurt am Main, Germany; ^13^Department of Pediatrics, Christian-Albrechts-University Kiel and University Medical Center Schleswig-Holstein, Kiel, Germany; ^14^Department of Pediatrics and Adolescent Medicine, Division of Pediatric Hematology and Oncology, University Hospital of Essen, Essen, Germany; ^15^Department of Pediatric Oncology, Hematology and Stem Cell Transplantation, Charité University Medicine Berlin, Berlin, Germany; ^16^Princess Máxima Center for Pediatric Oncology, Utrecht, The Netherlands; ^17^Department of Pediatric Hematology and Oncology, University Medical Center Hamburg-Eppendorf, Hamburg, Germany; ^18^Pediatric Hematology and Oncology, Hannover Medical School, Hannover, Germany; ^19^Pediatric Hematology/Oncology, Fondazione IRCCS Policlinico San Matteo, Pavia, Italy; ^20^Department of Human Genetics, Hannover Medical School, Hannover, Germany; ^21^German Cancer Consortium (DKTK), Heidelberg and Freiburg, Germany; ^22^Department of Pediatric Hematology and Oncology, IRCCS Ospedale Pediatrico Bambino Gesù, Sapienza, University of Rome, Rome, Italy; ^23^Current affiliation: Department of Pediatrics, University Hospital Salzburg, Paracelsus Medical University, Salzburg, Austria

**Introduction:** GATA2 deficiency is a heterogeneous multi-system disorder characterized by a high risk of developing myelodysplastic syndrome (MDS) and myeloid leukemia. Allogeneic HSCT is the only curative therapy for hematological complications of GATA2 deficiency; however, HSCT outcome for pediatric GATA2 patients remains to be fully elucidated.

**Aim:** The aim of the study was to characterize the HSCT outcome of pediatric patients with MDS and GATA2 germline mutations (GATA2^mut^) and compare it to MDS without GATA2 deficiency or any other known underlying predisposition (GATA2^wt^).

**Methods:** Sixty-five patients (34 males/31 females) reported to the registry of the European Working Group (EWOG) of MDS and treated with HSCT for GATA2-related MDS were diagnosed with RCC (36), MDS-EB (22), MDS-EBt (6) or MDR-AML (1) at a median age of 12.8 yrs. Karyotypes included monosomy 7 (44), der (1;7) (4), trisomy 8 (4), random aberration (1) or normal karyotype (12). Forty patients (71%) had additional non-hematological features of GATA2 deficiency. HSCT was performed from a matched sibling donor (17), unrelated donor (40) or mismatched family donor (8). Patients were prepared with busulfan-based (35), treosulfan-based (21), irradiation-based (5) or alternative conditioning regimens (4). For the analyses comparing GATA2^mut^ with GATA2^wt^ patients, 404 GATA2^wt^ MDS patients registered in EWOG-MDS in whom HSCT was performed during the same time period were identified.

**Results:** The probability of overall survival and disease-free survival (DFS) was 75% (63-87%) and 70% (58-82%), respectively. Non-relapse mortality (NRM) and relapse equally contributed to treatment failure (NRM 14% (8-26%), relapse incidence 16% (8-29%)). The cumulative incidence of acute graft-versus-host-disease (aGvHD) was 34% (24-48%) for grade II-IV and 12% (6-24%) for grade III-IV, while chronic GvHD occurred in 24% of patients at risk. There was no evidence of increased incidence of GvHD or excessive rates of infections or organ toxicities. Advanced disease and monosomy 7 (-7) were associated with worse outcome (DFS: RCC/-7 67%; MDS-EB/-7 69%; MDS-EBt/MDR-AML/-7 43%), whereas patients with RCC and normal karyotype showed an excellent outcome (DFS 90%). Comparing the outcome of GATA2^mut^ with GATA2^wt^ patients, there was no difference in DFS in patients with RCC and normal karyotype. Similarly, there was no significant difference among patients of any morphological subtype with monosomy 7 with respect to the presence or absence of GATA2 deficiency.

**Conclusions:** We demonstrate that pediatric patients with GATA2 deficiency are not at higher risk for HSCT-related complications or mortality compared to MDS patients without GATA2 germline mutations. Our results suggest that standard HSCT algorithms and protocols should be recommended for children and adolescents with GATA2 deficiency and MDS.

## 5. Clinical Characteristics, genomic landscape and functional studies of GATA2 deficiency in a cohort of Spanish patients

### D. Romero-Moya^1^, J. Castaño^2^, F. De Giorgio^1^, F. Lessi^3^, P. Aretini^3^, C.M. Mazzanti^3^, L. Mularoni^1^, M. Di Stefano^4^, A. Liquori^5^, J. Gonzalez^6^, E.J. Kozyra^7^, A. Catalá^4^, J.C. Rodriguez-Gallego^8^, J. Nomdedeu^9^, C. Díaz de Heredia^10^, A. Perez Martinez^11^, A. Escudero-Lopez^12^, F. López Cadenas^13^, M. Díez-Campelo^13^, T. Gonzalez^13^, C. Martínez-Laperche^14^, N. Dorado^14^, F. Marco^15^, J. Cervera^5^, C. Bodor^16^, M. Wlodarski^17^, A. Bigas^6^, A Giorgetti^1^, Grupo Español de Síndromes Mielodispásticos (GESMD)

#### ^1^Clinical Translational Program for Regenerative Medicine in Catalonia, Bellvitge Biomedical Research lnstitute (IDIBELL), L’hospitalet De Llobregat, Spain; ^2^Cellular and Advanced Therapy Unit, Banc de Sang i Teixits, Barcelona, Spain; ^3^Fondazione Pisana Per La Scienza ONLUS, Pisa, Italy; ^4^Servicio de Hematologia y Oncologia, Hospital Sant Joan de Deu, Barcelona, Spain; ^5^Hospital Universitari i Politècnic La Fe, Valencia, Spain; ^6^Cancer Research Program, Instituto Hospital del Mar de Investigaciones Médicas, Barcelona, Spain; ^7^University Childrens Hospital Freiburg, Freiburg, Germany; ^8^Servicio de Inmunología, Hospital Universitario de Gran Canaria Dr. Negrín, Las Palmas, Spain; ^9^Hematology Department, Hospital de la Santa Creu i Sant Pau, Universitat Autónoma de Barcelona, Barcelona, Spain; ^10^Pediatric Oncology and Hematology Department, Hospital Universitari Vall d’Hebron , Barcelona, Spain; ^11^Hospital Infantil Universitario Niño Jesús, Madrid, Spain; ^12^Molecular Pediatric Oncology Unit, Institute of Medical and Molecular Genetics (INGEMM), Hospital Universitario La Paz, Madrid, Spain; ^13^Departamento de Hematología, Hospital Clínico Universitario de Salamanca, Salamanca, Spain; ^14^Gregorio Marañón Health Research Insitute (IiSGM), Madrid, Spain; ^15^Biotechnología, Universidad de Alicante, Alicante, Spain; ^16^Department of Pathology and Cancer Research, Semmelweis University, Budapest, Hungary; ^17^Department of Hematology, St. Jude Children’s Research Hospital, Memphis, USA

##### **Correspondence:** D. Romero-Moya

**Introduction:** Germline heterozygous mutations in GATA2 are associated with a syndrome characterized by cytopenias, atypical infections, and increased risk of myelodysplastic syndrome (MDS) and acute myeloid leukemia (AML), often with an aggressive disease course and poor outcome. Penetrance and expressivity within GATA2 families is often variable, suggesting that additional aberrations are required to trigger the development of the disease. A mechanistic understanding of how GATA2 haploinsufficiency affects hematopoietic development and promotes myeloid malignant transformation is hindered by the limited number of cases reported and by the lack of appropriate human disease model system.

**Aim:** Unravel the molecular and cellular mechanisms of malignant progression of GATA2 deficiency.

**Methods:** We combined deep whole exome sequencing analysis (WES) with human induced pluripotent stem cells (hiPSCs) and hematopoietic stem cell-based disease models.

**Results**: Here we describe the genetic landscape and somatic changes of 13 Spanish GATA2-deficient patients using WES. Among the 13 GATA2 mutations funded half were novel (4/9). Median age at the diagnosis was 35 years (range 6-75 years) and clinical manifestation range from immunodeficiency, MDS and AML; one patient was asymptomatic at the time of genomic analysis, however in the last follow-up showed absolute monocytopenia and monosomy 7. WES analysis detected mutations in relevant genes involved in the NOTCH pathway and somatic mutations *in RUNX1*, *EZH2*, *ETV6, IDH2, TET2, GATA1* and *STAG2* as already reported in previous studies. Next, we studied the impact of two of the most recurrent germline GATA2 mutations associated with MDS (R396Q and R389W) using a human iPSC-based disease model. Applying a CRISPR/Cas9-mediated genome editing strategy we generated two hiPSC-GATA2 mutant lines and differentiated them toward blood progenitors. Data collected from FACS analyses showed that heterozygous GATA2 mutations (R396Q and R398W) lead to a block of early hematopoietic progenitor maturation (CD34+CD43+CD45+) and to an augment of myeloid progenitors’ compartment (CD33+/CD14+), which is in line with a low-risk MDS stage in childhood. The increased hematopoietic output of hiPSC-GATA2Mut lines could be related to the higher proliferation/survival of the emerging HPCs or CD33+cells. However, cell cycle analysis of CD34+ and CD33+ cells revealed no significant differences among hiPSC-GATA2^Mut^ and control. These data suggest a specific effect of GATA2 mutation on blood differentiation rather than on proliferation. Finally, we introduced GATA2^(R398W)^ mutation and selected somatic mutations (SETBP1 *GoF* and ASXL1 *LoF*) in healthy cord blood CD34+ cells by CRISPR/Cas9. Results from *in vivo* serial transplants of these cells into NSG-S mice suggest that germline GATA2 mutation alone is not sufficient to promote leukemia, however in combination with SETBP1+ASXL mutations a higher engraftment potential was observed in secondary transplants.

**Conclusions:** This study broadens the genomic and clinical portrait of a Spanish cohort of GATA2 carrier. Moreover, our results show that germline GATA2 R398W and R396Q induce an accumulation of early myeloid progenitors in early stage of hematopoietic development and reveal how hiPSC-based hematopoietic differentiation represents a promising disease model to study GATA2 deficiency. Collectively, our results help to increase our understanding of the molecular mechanism underlying GATA2 deficiency.

## 7. Pediatric myelodysplastic syndrome with inflammatory manifestations: Diagnosis, genetics, treatment, and outcome

### S. Barzilai-birenboim, A. Yanir, A. Krauss, J. Stein, O. Steinberg, S.O. Gilad, S. Noy Lotan, O. Dgany, T. Krasnov, Y. Kodman, T. Feuerstein, J. Mardoukh, H. Fishman, I. Geron, Dr Joanne Yacobovich, S.Barzilai-Birenboim, Yehudit, S Izraeli

#### Schneider Children’s Medical Center Of Israel, Petah-Tikva, Israel

##### **Correspondence:** A.Yanir

**Introduction:** Inflammatory manifestations (IM) are well described in adult patients with myelodysplastic syndrome (MDS), but the presentation is highly variable, and no standardized treatment exists. This phenomenon is rarely reported in children. As more pediatric patients are hematopoietic stem cell transplantation (HSCT) candidates, the role of anti-inflammatory treatment in relation to HSCT should be defined.

**Aim:** We report a series of five children from a tertiary center, describe the clinical presentation, molecular findings, and treatment options.

**Methods/Results:** All patients presented with advanced MDS with blast percentages ranging 10–30%, all had severe IM. One patient had MDS secondary to severe congenital neutropenia, the other four patients had presumably primary MDS. All four were found to harbor a PTPN11 gene driver mutation, which is found in 35% of cases of juvenile myelomonocytic leukemia. The mutation was present in the myeloid lineage but not in T lymphocytes. Three children had symptoms of Behcet’s-like disease with trisomy 8 in their bone marrow. All patients were treated with anti-inflammatory medications (mainly systemic steroids) in an attempt to bring them to allogeneic HSCT in a better clinical condition. All demonstrated clinical improvement as well as regression in their MDS status post anti-inflammatory treatment. All children have recovered from both MDS and their inflammatory symptoms post HSCT.

## 9. Germline SAMD9L Triple-Allelic Mosaicism with Independent Segregation into Biallelic Offsprings: Molecular Support for Non-Mendelian Inheritance

### S. Sahoo^1^, C.Goodings^1^, M. Angeles Lillo^1^, T.Lammens^2^, J. van der Werff ten Bosch^3^, B.Strahm^4^, B. De Moerloose^2^, M.Wlodarski^1^

#### ^1^Department of Hematology, St. Jude Children’s Research Hospital, Memphis, United States; ^2^Department of Paediatric Haematology-Oncology, Ghent University Hospital, , Belgium; ^3^Department of Pediatric Hematology Oncology, University Hospital Brussel, , Belgium; ^4^Department of Pediatrics and Adolescent Medicine, Division of Pediatric Hematology and Oncology, Medical Center, Faculty of Medicine, University of Freiburg, Germany

##### **Correspondence:** S. Sahoo

**Introduction/Aims:** Single nucleotide variants (SNV) in humans are mostly biallelic; however, multiallelic variation (multiple different sequence variants observed at the same site) are estimated to affect ~8% of all SNV sites. Known examples of multiallelic variation are the ABO and HLA systems. Despite multiple alleles in population, only two alleles are present at the same time in one individual, unless a chromosomal triploidy occurred. Here, building on the discovery of a family with triple allelic mosaicism at a single *SAMD9L* nucleotide position we aimed to decipher the underlying genetics and model the mutations *in vitro* and *in vivo*.

**Methods:** We applied genomic methods (Sanger, whole exome sequencing, error-corrected deep sequencing, single-cell DNA sequencing (scDNAseq), digital-droplet PCR and TA-cloning), developed mutant HEK293 cell lines (via stable integration of mutant cDNA using piggyBac transposon system) and mutant inducible pluripotent stem cells (iPSC) using CRISPR-Cas9 editing, and created mouse models with constitutive *Samd9L* mutations.

**Results:** We present a family with 2 germline *SAMD9L* mutations, where the index female patient carried heterozygous c.4534G>A (p.V1512M) mutation and presented with severe pancytopenia, del7q and marrow findings consistent with aplastic anemia, while the brother carried heterozygous c.4534G>T (p.V1512L) mutation and had refractory cytopenia with a milder clinical course. The asymptomatic mother who had normal diploid karyotype was found to be a mosaic for all 3 alleles (wildtype and 2 mutations), with wildtype allele present at ~50% frequency in all tissues, while both mutant alleles showing different distribution depending on tissue origin (hematopoiesis: p.V1512M at ~9% and p.V1512L at 41%, hair follicles: both at ~25%). To understand why the mother had no abnormal phenotype, we performed scDNAseq in peripheral blood and identified UPD7q as a rescue event present in 18% of cells. We next modeled the mutations *in vitro*. Both mutations caused enhanced growth arrest in HEK293T cells, and hematopoietic differentiation of mutant iPSC yielded decreased CD45+CD18+ myeloid cells, with V1512M showing statistically more severe phenotype compared to V1512L in both cellular systems. Mice with heterozygous *Samd9L* V1512M mutation showed embryonic lethality exceeding 50% occurring between E9.5-E11; most newborns died by the age of 2weeks. Histopathological characterization of V1512M mice revealed hematologic (B-cell lymphopenia, anemia) and constitutional abnormalities (runted-growth, hydronephrosis, degeneration of thymus and ovary). In contrast, V1512L mice had no baseline phenotype. We generated a compound heterozygous intercross (V1512M/V1512L). These mice were active for 2months but begun dying between age 19 and 25weeks. The phenotype was similar (albeit milder) to V1512M mice. Further analysis of hematopoiesis in V1512L mice after different conditions (aging, chemical stress), as well as detailed characterization of marrow findings in V1512M/V1512L intercross, and survival analyses are underway.

**Conclusions**: Our study establishes divergent phenotypic effects of a single amino acid substitution in *SAMD9L* in human, mice, and cell lines. To our knowledge, this is the first report of germline triple-allelic mosaicism with bi-allelic segregation. The mosaicism is likely a result of an unsuccessful repair of the highly deleterious V1512M mutation during early embryogenesis in the mother. Another explanation for the germline tri-allelic state deals with the rare phenomenon of dispermic fertilization.

Consent to publish has been obtained.

## 11. Cellular and metabolic characteristics of pre-leukemic hematopoietic progenitors with GATA2 haploinsuficiency

### A. Rein^1,2,3^, I. Geron^1,2,3,14^, E. Kugler^1,2,3^, H. Fishman^1,2,3^, E. Gottlieb^4^, I. Abramovich^4^, A. Giladi^5^, I. Amit^5^, R. Mulet- Lazaro^6^, R. Delwel^6,7^, S. Gröschel^6,8,9^, S.Levin-Zaidman^10^, N. Dezorella^10^, V. Holdengreber^11^, T. Nageswara Rao^12^, J. Yacobovich^2^, O. Steinberg-Shemer^2,14^, Q.-H. Huang^13^, Y.Tan^13^, S.-J. Chen^13^, S. Izraeli^1,2,3,14^ and Y. Birger^1,2,3,14^

#### ^1^Department of Human Molecular Genetics and Biochemistry, Sackler Medical School, Tel Aviv University, Tel Aviv 69978, Israel; ^2^The Rina Zaizov Division of Pediatric Hematology-Oncology, Schneider Children’s Medical Center, Petah Tikva; Israel; ^3^Functional Genomics and Childhood Leukaemia Research, Sheba Medical Centre, Tel-Hashomer, Israel; ^4^Technion Integrated Cancer Center, Faculty of Medicine, Technion Israel Institute of Technology, Haifa, Israel; ^5^Department of Immunology, Weizmann Institute of Science, Rehovot, Israel; ^6^Department of Hematology, Erasmus University Medical Center, Rotterdam, 3015 GE, the Netherlands; ^7^Oncode Institute, Erasmus University Medical Center, Rotterdam, the Netherlands; ^8^Molecular Leukemogenesis, Deutsches Krebsforschungszentrum, 69120 Heidelberg, Germany; ^9^Department of Internal Medicine V, Heidelberg University Hospital, Heidelberg, Germany; ^10^Electron Microscopy Unit, Weizmann Institute of Science, Rehovot, Israel; ^11^Electron Microscopy Unit, IDRFU, Faculty of Life Sciences, Tel Aviv University, Israel; ^12^Stem Cells and Leukemia Laboratory, University Clinic of Hematology & Central Hematology, Department of Biomedical Research (DBMR), Inselspital Bern, University of Bern, Switzerland; ^13^State Key Laboratory of Medical Genomics, Shanghai Institute of Hematology, Rui Jin Hospital, Jiao Tong University School of Medicine, Shanghai 200025, China; ^14^Felsenstein Medical Research Center, Sackler School of Medicine Tel-Aviv University, Petah Tikva; Israel

Mono-Allelic germline disruptions of the transcription factor GATA2 result in a propensity for developing myelodysplastic syndrome (MDS) and acute myeloid leukemia (AML). How a partial loss of GATA2 enables leukemic transformation occurring years later in life, is unclear. This question is unsolved mainly as Gata2 heterozygote mice do not develop hematologic malignancies. Here we show that two different germline Gata2 mutations accelerate AML in mice expressing the human hematopoietic stem cell regulator ERG. Analysis of ERG/Gata2^**het**^ fetal liver and bone marrow derived hematopoietic cells revealed a distinct pre-leukemic phenotype characterized by enhanced transition from stem to progenitor state, increased proliferation, and a striking mitochondrial phenotype, consisting of highly expressed Oxidative-Phosphorylation related gene-sets, elevated oxygen consumption rates, and markedly distorted mitochondrial morphology. Importantly, the same mitochondrial gene-expression signature was observed in human AMLs harboring GATA2 aberrations. Similar to the observations in mice, non-leukemic bone marrows from children with germline GATA2 mutation demonstrated marked mitochondrial abnormalities. Thus, we observed the tumor suppressive effects of GATA2 in two germline Gata2 genetic mouse models. As oncogenic mutations often accumulate with age, Gata2 deficiency mediated priming of hematopoietic cells for oncogenic transformation may explain the earlier occurrence of MDS/AML in patients with GATA2 germline mutation. The mitochondrial phenotype is a potential therapeutic opportunity for prevention of leukemic transformation in these patients.

## 12. Germline GATA2 mutations in familial MDS/AML: a manually curated online registry from the ERAPERMED GATA2-HuMo international consortium

### L. Kotmayer^1^, E. Kozyra^2^, A. Bekő^1^, T. László^1^, A. Bigas^3^, A. Giorgetti^4^, K. Kállay^5^, M. Wlodarski^2,6^, C. Bödör^1^

#### ^1^HCEMM-SE Molecular Oncohematology Research Group, 1st Department of Pathology and Experimental Cancer Research, Semmelweis University, Budapest, Hungary; ^2^Division of Pediatric Hematology and Oncology, Department of Pediatrics and Adolescent Medicine, Medical Center, Faculty of Medicine, University of Freiburg, Germany; Faculty of Biology, University of Freiburg, Freiburg, Germany; ^3^Cancer Research Program, Institut Hospital del Mar d’Investigacions Mèdiques, CIBERONC, Hospital del Mar, Barcelona, Spain; ^4^Regenerative Medicine Program, Bellvitge Institute for Biomedical Research (IDIBELL) and Program for Clinical Translation of Regenerative Medicine in Catalonia (P-CMRC), 08908 L’Hospitalet del Llobregat, Spain; ^5^Pediatric Hematology and Stem Cell Transplantation Department, Central Hospital of Southern Pest – National Institute of Hematology and Infectious Diseases; ^6^Department of Hematology, St. Jude Children’s Research Hospital, Memphis, USA

**Introduction:** Pathogenic variants of the GATA2 transcription factor are one of the most common predisposing mutations of familial myeloid malignancies, including acute myeloid leukaemia (AML) and myelodysplastic syndrome (MDS). *GATA2*-associated familial AML/MDS is frequently characterized by recurrent or atypical bacterial infections and lymphedema, but despite of the distinctive clinical manifestations, AML/MDS with *GATA2* predisposition may go unrecognized due to incomplete penetrance and missing family history. Timely diagnosis is often further complicated by the lack of clear phenotype-genotype correlations. Although during the past few years a large number of *GATA2* mutations have been identified by genetic testing, the number of sizeable cohort studies are still limited, making the clinical interpretation of these variants challenging.

**Aim:** Our aim was to develop an online, public database of germline *GATA2* mutations and associated clinical phenotypes based on international publications to aid the clinical recognition of patients harboring predisposing variants. With this database we also aim to scrutinize phenotype-genotype correlations based on pre-selected histopathological and clinical features.

**Methods:** Familial AML/MDS cases with germline variants from 87 case reports and cohort studies published between 2009-2021 were identified via PubMed search and literature review. PubMed search was performed using a combination of keywords ‘GATA2’, ‘deficiency’, ‘mutation’, ‘familial’, ‘acute myeloid leukaemia’, ‘myelodysplastic syndrome’, ‘Emberger-syndrome’ and ‘MonoMac’. Individual cases were uploaded to a self-developed online database, where clinical and pathological findings were classified based on the involvement of different organ systems. Accurate data submission was ensured by manual data entry and curation. Data visualization was performed using the Microsoft Power BI software.

**Results:** To date, 412 cases with germline *GATA2* variants were collected and uploaded to the database. Familial AML and MDS were described in 13.1% (54/412) and 56.8% (234/412) of the patients, respectively. Predisposition syndromes, such as Emberger-syndrome or MonoMac were identified in 10.2% (42/412) of the cases. Nearly one-quarter (24.3%, 100/412) of the cohort had no symptoms associated with hematologic malignancies highlighting the incomplete penetrance of *GATA2* predisposition. Clinical and histopathologic findings of symptomatic carriers were classified into 15 main categories including hematologic and immunologic features, lymphatic disorders, respiratory tract disorders and other malignancies. Twenty-six further subcategories allow for a more accurate data filtering. In total, 1,484 records are now included in the hierarchically structured dataset with a median 3 (range: 0-16) findings per case. After excluding overt myeloid malignancies, bacterial infections were found to be the most common symptoms of GATA2 deficiency. Most recurrent variants of *GATA2* were T354M and R396Q, but further analyses are required to assess phenotype-genotype correlations.

**Conclusions:** Although recent studies have described a large number of germline *GATA2* variants, assessment of the mutations’ clinical significance is still an unmet need in the management of patients with familial AML/MDS. Here we developed the first online interactive, public database for the research of phenotype-genotype correlations in this entity. Manual curation and statistical analyses are still ongoing, preliminary release of the GATA2-HuMo germline *GATA2* registry is expected by the end of 2021.

## 13. Leukemogenesis in GATA2 haploinsufficient mice is a secondary event after bone marrow failure

### J. Fernandez-Orth^1^, J.M. Weiss^1^, G. Andrieux^2^, V.R. Mittapalli^1^, S. Bohler^1^, C. Molnar^1^, C. Frank^1^, I. Gonzalez-Mendez^3^, B. Strahm^1^, D. Steinemann^4^, L. Quintanilla-Martinez^3^, M. Börries^2^, C. Niemeyer^1^ and M. Erlacher^1^

#### ^1^University Medical Center Freiburg, Center for Pediatrics Department of Pediatric Hematology and Oncology, Freiburg, Germany; ^2^Institut für Medizinische Bioinformatik und Systemmedizin Medizinische Fakultät, Albert-Ludwigs-Universität Freiburg; ^3^University of Tuebingen, Institute of Pathology and Neuropathology, Tuebingen, Germany; ^4^Hannover Medical School, Human Genetics Department, Hannover, Germany

**Introduction:** Hematopoiesis is a tightly regulated process where transcription factors and cofactors ensure the proper proliferation, differentiation and survival of immature hematopoietic progenitor cells. Mutations in these genes can affect not only their expression levels but also their activity, disturbing hematopoiesis and leading to a high risk of developing bone marrow failure and/or leukemia. One important transcription factor governing the hematopoietic system is GATA2, which plays an essential role in the hematopoietic stem and progenitor cell (HSPC) development and differentiation. GATA2 belongs to the GATA transcription factor family and controls gene transcription from multiple target genes by binding to a consensus DNA sequence (of many enhancers, suppressors and promotors. More than 400 mutations were already described in GATA2, leading to a broad phenotypic spectrum in patients. While the hematological and non-hematological phenotypes caused by GATA2 haploinsufficiency are very variable, the risk of developing either MDS or AML is exceedingly high. However, it is still unknown whether leukemia emerges as a primary event or however, as a consequence of BM failure. Despite this high risk, the mechanisms underlying malignant transformation are poorly described. So, in order to predict, treat and prevent leukemic transformation in affected individuals, it is necessary to further understand the mechanisms underlying GATA2 syndrome. We hypothesized that GATA2-associated leukemia does not develop as a primary event but rather occurs as a secondary event after BM failure.

**Methods/Results:** Using mice with a GATA2 haploinsufficient hematopoietic system, we generated a mouse model which developed leukemias spontaneously. We could observe that leukemias developed spontaneously after stem and progenitor cell transplantation into lethally irradiated WT hosts. About 15% of recipient mice succumbed due to leukemia. However, leukemias emerged exclusively in mice that developed bone marrow failure first (40% of all recipients). In contrast, 60% of recipients remained healthy. While no somatic alterations were detected in mice suffering from bone marrow failure alone, leukemia development was always associated with somatic mutations and/or chromosomal aberrations, indicating clonal evolution. These somatic aberrations were overlapping with those occurring in humans with GATA2 syndrome. Analysis of stem and progenitor cells isolated from recipient mice at early time points (i.e. prior development of bone marrow failure) showed profound transcriptomic changes, indicating that beside genetic alterations, epigenetic alterations might contribute to the different outcomes in our mouse model.

**Conclusion:** We could conclude that GATA2 haploinsufficiency predisposes to bone marrow failure in mice, paving the way for secondary leukemia.

## 14. The 6-year experience on childhood myelodysplastic syndromes of the Greek study group for MDS/JMML/SAA, as a member of the EWOG-MDS/SAA working group

### L. Petrikkos^1^, K. Stefanaki^2^, N. Tourkantoni^3^, E. Dikaia Ioannidou^4^, K. Tsitsikas^1^, K. Manola^5^, A. Bountali^1^, M. Kourti^6^, K. Antoniadi^1^, H.Tsipou^3^, E.Dana^7^, I. Pelagiadis^8^, A. Tragiannidis^9^, E. Mantadakis^10^, M. Economou^11^, M. Servitzoglou^12^, A. Makis^13^, E. Papakonstantinou^6^, E. Chatzipantelis^9^, H. Kosmidis^7^, E. Stiakaki^8^, M. Baka^12^, C. Kelaidi^1^, G. Paterakis^14^, E. Goussetis^4^, A. Kattamis^3^, I Peristeri^4^, S.Polychronopoulou^1^

#### ^1^Department of Paediatric Haematology-Oncology (T.A.O.), «Aghia Sophia» Children’s Hospital, Athens, Greece; ^2^Department of Pathology, «Aghia Sophia» Children’s Hospital, Athens, Greece; ^3^Division of Paediatric Hematology-Oncology, 1st Dpt Of Pediatrics, National & Kapodistrian University Of Athens, «Aghia Sophia» Children’s Hospital, Athens, Greece; ^4^Bone Marrow Transplantation Unit, «Aghia Sophia» Children’s Hospital, Athens, Greece; ^5^Laboratory of Health Physics, Radiobiology and Cytogenetics, National Center for Scientific Research «Demokritos», , Athens, Greece; ^6^Department of Paediatric Oncology, Hippokration General Hospital, Thessaloniki, Greece; ^7^Pediatric & Adolescent Oncology Clinic, “Mitera” Hospital, Athens, Greece; ^8^Department of Paediatric Hematology-Oncology, University Hospital of Heraklion, Heraklion, Crete, Greece; ^9^Division of Paediatric & Adolescent Hematology-Oncology, 2nd Dpt of Paediatrics, Aristotle University of Thessaloniki, AHEPA General Hospital, Thessaloniki, Greece; ^10^Department of Pediatrics, Democritus University of Thrace, University General Hospital of Alexandroupolis, Alexandroupolis, Greece; ^11^Division of Haematology, 1st Dpt of Paediatrics, Aristotle University of Thessaloniki, Hippokration General Hospital, Thessaloniki, Greece; ^12^Oncology Department, “P&A Kyriakou” Children’s Hospital, Athens, Greece; ^13^Department of Paediatrics, University of Ioannina, University Hospital, Ioannina, Greece; ^14^Immunology Department, Flow Cytometry Laboratory, “G.Gennimatas” General Hospital, Athens, Greece

##### **Correspondence:** L. Petrikkos

**Introduction/Aim:** Myelodysplastic syndromes (MDS) in childhood consist in a heterogeneous group of clonal hematopoietic disorders often associated with dysmorphic characteristics, genetic and MDS-predisposition syndromes. We present the 6-year experience on MDS, of the Greek study group for MDS/JMML/SAA, as a member of the EWOG MDS/SAA European working group.

**Methods:** Thirty-two (32) MDS patients diagnosed during the period 10/2015 – 7/2021 are presented. Appropriate detailed clinical, laboratory, cytogenetic and pathology investigations were performed. Diagnostic samples and clinical data were reviewed in national reference centers. The patients were treated in the paediatric hematology-oncology departments and the pediatric BMT-unit of Greece, and were consequently registered to EWOG-MDS.

**Results:** The 32 patients (average age: 9,7 years, 20/32 male) were classified according to EWOG-criteria as following: i) Refractory Cytopenia of Childhood (MDS-RCC) 27/32 (2 out of 27 were secondary post chemotherapy due to prior malignancy), ii) MDS with excess blasts (MDS-ΕΒ) 5/32. At diagnosis: median WBC 3435/μl, median Hb 8.8 gr/dl and median PLTs 38000/μL. The bone marrow smear morphology at diagnosis revealed dysplasia of one/two/three hemopoietic lineages in 2/7/23 patients respectively. Seven patients presented with dysmorphic features or/and genetic stigmata (mainly in face and extremities). Cytogenetics: monosomy 7 in 3/32 patients (all RCC), trisomy 8 in 2/32 and inv17 in 1/32. The three patients with monosomy 7 and the one with inv17 , all RCC, were positive (4/32) for *SAMD9/SAMD9L* mutations. Twelve (12/32) patients are under watchful waiting (one of twelve with monosomy 7 and *SAMD9L* mutation), 18/32 underwent stem cell transplantation (SCT) due to MDS-ΕΒ (5/18), monosomy 7 (2/18), inv17 (1/18), or increase in transfusion needs (10/18 with MDS-RCC), while in 2/32 SCT is pending. Mean time from diagnosis to SCT was 5,5 months. Thirty (30/32) patients are alive (OS 94%). Out of the 18 transplanted patients 14 are in remission without transfusion needs, 1 is in remission after 2nd SCT (due to relapse) and 1 in search for a new donor after graft rejection. Two patients died, one due to refractory disease after second SCT and one due to severe infection and cGvHD.

**Conclusions:** MDS in childhood pose difficulties for accurate diagnosis and appropriate treatment. Participation in national collaborative groups and scientifically established international working groups such as EWOG-MDS/SAA contributes: a) to understand in depth the pathophysiology of diseases, b) to clarify, on the basis of molecular, genetic and clinical criteria, which patients benefit from SCT and which do not, and c) to investigate the optimal SCT timing or other appropriate therapeutic approach, at a time.

## 15. Favorable outcome for young patients receiving allogeneic stem cell transplantation for RUNX1 germline mutation related diseases

### B.Strahm^1^, G. Lucchini^2^

#### ^1^Paediatric Haemato-Oncology Unit, University of Freiburg, Freiburg, Germany; ^2^Great Ormond Street Hospital NHS Foundation Trust, London, United Kingdom

##### **Correspondence:** B.Strahm

**Introduction:** Autosomal dominant mutations in RUNX1 gene are rare and have been associated to a familiar platelet disorder with a 35% risk of progression to acute myeloid leukemia (AML) in a lifetime and median age at transformation of 33 years. Haematopoietic stem cell transplantation (HSCT) is the treatment of choice for these patients, and a higher risk of relapse post SCT has been described in adults with RUNX1 related AML. Families harbouring specific mutations seem to be more prone to develop AML and a degree of anticipation is observed in pedigrees. Children who harbour a germ line mutation represent a yet to be studied group of patients, for which risk of malignant evolution as well as outcomes and risks of haematopoietic stem cell transplantation (HSCT) are unknown.

**Aims:** This multicentre retrospective study describes HSCT characteristics and clinical outcomes of patients diagnosed with RUNX1 germline mutations during childhood who developed haematological diseases (from isolated thrombocytopenia to AML) and underwent HSCT by the age of 20.

**Methods/Results:** We identified 25 patients from 23 families and 9 European countries meeting inclusion criteria. Median age at diagnosis was 7.9 years. 15 patients had a known familiar history of thrombocytopenia/RUNX1 related disease. At diagnosis 11/25 patients presented with thrombocytopenia or refractory cytopenia of childhood (RCC). With time 2/11 progressed to myelodysplasia with excess of blasts (MDS-EB) and 2/11 transformed into AML. 10/25 patients had MDS-EB at diagnosis, 2/10 progressed to MDS EB in transformation (MDS-EBt) and 2/10 to AML. For 5/25 patients AML was the presenting diagnosis. 4/25 patients had a RUNX1 mutation detected retrospectively after HSCT. 16 patients had normal karyotype, 2 had monosomy 7, 1 each had trisomy 8, deletion 5q, t(8;12) and a hyperdiploid karyotype. 2 patients (1 AML, 1MDS EBt ) received azacytidine, 8 patients (5 AML and 2 MDS-EB) received a combination of chemotherapy regimen or chemotherapy and azacytidine before HSCT. At the time of HSCT 5/8 patients who had developed AML were not in remission. Patients received their HSCT from MSD (5), MUD (12), MMUD (7) and MMFD (1). Bone marrow was the favourite source of stem cells (15/25) , followed by peripheral blood (7/25) and cord blood (3/25). 22/25 patients received a myeloablative conditioning regimen, which was busulfan (14) treosulfan (6) or total body irradiation based (2). 16/25 patients received serotherapy as part of the conditioning regimen. There were no primary graft failures. One patient diagnosed with MDS-EB died from transplant related mortality on day +27 (combination of severe hyperacute graft versus host disease and veno occlusive disease), 22/25 pts are alive with a median follow up of 3.5 years. Of note 4/5 patients who presented AML not in remission prior to HSCT (median blasts prior to SCT 25%) achieved a complete continuous remission following HSCT. Three patients relapsed following HSCT and underwent a second HSCT. One is alive in CR, 1 died from further disease relapse and 1 from transplant related mortality following second SCT.

**Conclusion:** Stem cell transplantation is well tolerated in children with RUNX 1 germline mutation and outcomes are favourable even in patients transformed to AML. Natural history of the disease with identification of patients who are more prone to transform during the paediatric age will be important to select candidates for pre-emptive approach.

## 16. Clinical application of transactivation assays for precise classification of germline RUNX1 missense variants associated with predisposition to MDS and AML

### M. Decker^1^, A. Agarwal^2^, A. Benneche^3^, J.Churpek^4^, N. Duployez^5^, A.S. DuVall^6^, M. P.T. Ernst^7^, A. Förster^1^, H. Høberg-Vetti^3,8^, M. Nash^9^, M.H.G.P. Raaijmakers^7^, T.H.A. Tvedt^10^, A. Vlachos^9^, B. Schlegelberger^1^, T. Illig^1,11^, T. Ripperger^1^

#### ^1^Department of Human Genetics, Hannover Medical School, Hannover, Germany; ^2^Division of Hematology and Medical Oncology, Oregon Health & Science University Knight Cancer Institute, Portland, USA; ^3^Western Norway Familial Cancer Center, Department of Medical Genetics, Haukeland University Hospital, Bergen, Norway; ^4^Department of Medicine, Section of Hematology, Oncology, and Palliative Care, The University of Wisconsin-Madison, Madison, USA; ^5^Department of Hematology, CHU Lille, University Lille, INSERM U1277, Lille, France; ^6^Division of Medicine, Section of Hematology/Oncology, University of Chicago, Chicago, USA; ^7^Department of Hematology, Erasmus MC Cancer Institute, Rotterdam, The Netherlands; ^8^Affiliated Partner of the European Reference Network on Genetic Tumour Risk Syndromes, ERN GENTURIS, Project ID No 739547; ^9^Division of Hematology/Oncology and Cellular Therapy, Cohen Children’s Medical Center, Northwell Health, New York, USA; ^10^Department of Medicine, Haukeland University Hospital, Bergen, Norway; ^11^Hannover Unified Biobank, Hannover Medical School, Hannover, Germany

##### **Correspondence:** M. Decker

**Introduction:** Familial platelet disorder with associated myeloid malignancies (RUNX1-FPD, FPDMM, FPD/AML, MIM 601399) is typically characterized by thrombocytopenia, functional platelet defects, and a ~40% risk to develop hematological malignancies, mainly MDS and/or AML. Leukemic transformation requiring secondary somatic events can occur from early childhood to late adulthood. RUNX1-FPD is caused by heterozygous pathogenic germline variants in RUNX family transcription factor 1 *(RUNX1)*. Based on present gene variant classification guidelines, rare *RUNX1* missense variants must frequently be classified as variants of uncertain significance (VUS).

**Aim:** To overcome this challenge, we have previously reported the development and application of a set of assays to characterize the functional impact of RUNX1 variants addressing its heterodimerization with CBFB, its phosphorylation, and its ability to activate transcription. In the present study, we evaluated the applicability of our transactivation assays to investigate RUNX1 variants in different regions of the protein by studying a set of nonsense variants distributed from the N- to the C-terminus of the protein. In addition, the transactivation assays were applied to investigate 11 *RUNX1* variants detected in patients with thrombocytopenia, MDS, AML, or as secondary finding to independently validate our assays.

**Methods:** We analyzed the ability of RUNX1 variants to activate transcription of RUNX1 target genes (i.e., *CSF1R*, *ETV1*, and *MYL9*) by applying luciferase reporter assays in HEK293T and HEL cells. As controls, wild-type (WT) RUNX1b, the known pathogenic variant Arg139Gln, and the benign variant Leu29Ser were investigated in parallel. Functional data were integrated in ACMG/AMP variant classification following the current expert guidelines of the ClinGen Myeloid Malignancies variant curation expert panel (MM-VCEP).

**Results:** We showed that the majority of RUNX1 missense variants detected in RUNX1-FPD can functionally be addressed by our transactivation assays. Following the MM-VCEP expert guidelines, two VUS were reclassified to likely pathogenic and analyses supported the (likely) pathogenic classification of two additional variants. We demonstrated functionality of four VUS, but reclassification to (likely) benign was not possible following current classification guidelines. Hence, RUNX1-FPD suspicion was confirmed in three families with RUNX1-FPD-specific medical history. For variants identified in non RUNX1-FPD-typical families, no functional defects were observed.

**Conclusions:** In the present study investigating 11 variants, the clinical utility of our transactivation assays was independently validated and illustrated in the context of seven index patients. Even though most missense variants detected in the context of RUNX1-FPD can functionally be addressed by our transactivation assays, additional assays are required to investigate C-terminal RUNX1 missense variants. Careful reevaluation of current variant classification guidelines should be considered to allow future reclassification of VUS to (likely) benign, if sufficient evidence for its functionality was observed. Applying functional assays supports final *RUNX1* variant classification and can be essential for precise genetic diagnosis. It facilitates translation of genetic data into personalized medical care for index patients and their relatives who are at risk.

## 17. GATA2 in lineage differentiation and stem cell fitness

### E. Gioacchino, C. Koyunlar, H. de Looper, J. Peulen, D. Bosch, R. Hoogenboezem, P. van Strien, E. Bindels, K. L Gussinklo, E. Dzierzak^1,2^, I. Touw and E. de Pater

#### ^1^Department of Hematology, Erasmus MC, Rotterdam, The Netherland; s^1^Dept of Cell Biology, Erasmus MC, Rotterdam, The Netherlands; ^2^The Queen’s Medical Research Institute College of Medicine and Veterinary Medicine, Edinburgh, United Kingdom

MonoMAC and Emberger syndromes are GATA2 haploinsufficiency syndromes. These syndromes are characterized by monocytopenia, neutropenia, B-, dendritic- and NK cell lymphopenia and up to 80% of patients with innate GATA2 mutations develop myelodysplastic syndrome (MDS)/ acute myeloid leukemia (AML). Gata2 level is a key factor in embryonic hematopoietic stem cell (HSC) generation and maintenance. To study the effect of Gata2 haploinsufficiency on the mechanism of malignant transformation, we use both mouse and zebrafish Gata2 haploinsufficiency models. Gata2 heterozygous mutant mice survive to adulthood without developing any visible bone marrow phenotype but their phenotypic HSCs are lower in frequency, more proliferative and transcriptionally more committed to differentiation compared to WT. Furthermore, they show increased DNA damage indicating these HSCs experience proliferative stress. Upon aging and transplantation these HSCs repopulate the bone marrow, but are impaired in their contribution to hematopoiesis resulting in B-cell lymphopenia.

In zebrafish, two orthologues of *Gata2* exist: *gata2a* and *gata2b, of which lineage analysis revealed that all hematopoietic cells once expressed Gata2b.* We found that complete loss of *gata2b* results in a severe reduction in myeloid differentiation. Furthermore, *gata2b*^*+/-*^ zebrafish develop dysplasia of both erythroid and myeloid lineages as is found in patients with GATA2 mutations. Using single-cell RNA sequencing we aim to identify early onset markers of dysplasia and unveil the mechanism of malignant transformation.

Our mouse and zebrafish models recapitulate characteristics of the GATA2 deficiency syndromes and it can give new insight into the pathophysiology of this disease.

## 18. T lymphocyte profile and dynamics in patients with hepatitis-associated bone marrow failure

### M. Novakova^1#^, M. Svaton^1#^, A. Skotnicova^1^, M. Sukova^2^, D. Pospisilova^3^, O. Fabri^4^, P. Svec^4^, J. Trka^1^, O. Hrusak^1^, J. Stary^2^, E. Mejstrikova^1#^, E. Fronkova^1#^

#### ^1^CLIP - Childhood Leukaemia Investigation Prague, Department of Paediatric Haematology and Oncology, Second Faculty of Medicine, Charles University and University Hospital Motol, Prague, Czech Republic; ^2^Department of Paediatric Haematology and Oncology, Second Faculty of Medicine, Charles University and University Hospital Motol, Prague, Czech Republic; ^3^Department of Pediatrics, Palacky University and University Hospital Olomouc; ^4^Department of Pediatric Hematology and Oncology, National Institute of Children’s Diseases and Medical Faculty, Comenius University, Bratislava, Slovakia

#These authors contributed equally.

**Introduction:** Hepatitis-associated bone marrow failure (HABMF) is a rare variant of bone marrow failure, which is typically diagnosed 2-3 months before failure itself after attack of hepatitis in patients that are seronegative for known hepatitis viruses. Although very little is known about its etiology, an autoimmune mechanism is presumed based on clinical response to immunosuppressive therapy (IST) and reports of oligoclonal T-lymphocyte expansion and skewing of CDR3 region length.

**Aim:** Our aim was to uncover the role of T cell lymphocytes in pathogenesis by analysis of their mature stage, activation and T cell receptor beta repertoire (TRBV).

**Methods:** We included 21 pediatric patients diagnosed with HABMF in Czech Republic in 2004-2021 and 1 patients diagnosed in Slovakia. The prevalence of HABMF in pediatric patients with BM failure in Czech Republic in this period (n=121; excluding patients with known genetic cause) was 17%. Interestingly, 5 out of total 22 HABMF patients had histopathological findings consistent with refractory cytopenia of childhood (RCC). We performed flow cytometry immunophenotyping in bone marrow (BM) and peripheral blood (PB) samples at diagnosis and at day 120 after initiation of immunosuppressive therapy (IST). TRB sequencing was performed following the SOP developed by the EuroClonality-NGS working group with DNA input normalized to the equivalent of 20 000 CD3+ cells per sample based on flow cytometry data. Bioinformatic analysis was performed with ARResT/Interrogate. WES was performed on Illumina NextSeq 500 and libraries prepared using the Agilent SureSelectXT Human All Exon V6+UTRs kit.

**Results:** We found significant activation of CD8pos T cells based on the expression of HLA-DR and a decrease of naive forms CD27posCD45RApos compared to non-HAAA RCC and AA patients. TRB repertoire analysis revealed expanded clones (>5%) of T lymphocytes in 8 diagnostic samples. The CDR3 sequences of expanded clones differed among patients. In one HABMF patient the expanded clone comprised 39% of all TRBV sequences and immunophenotypically belonged to CD8pos cells with highly abundant senescent CD57pos T cells. WES analysis did not reveal any previously published variant in genes associated with immune dysregulation, immunodeficiency or bone marrow failure. Rare (0.01% in gnomAD) heterozygous variants of unknown significance were found in *ACD* (in 2 patients), *ASXL1*, *CXCR4, IFIH1* and *SOS1* genes. Sixteen patients were treated according to standard European Working Group on MDS/SAA protocols with anti-thymocyte globulin (horse n=7; rabbit n=9), corticosteroids and cyclosporin A. Eight patients reached complete remission on IST by day 120 and 8 patients with insufficient response underwent MUD-HSCT. We did not observe any correlation of T cell populations at diagnosis or day 120 of IST (activated and exhausted T cells) and clinical response. Similarly, the presence of an immunodominant clone or oligoclonal TRB repertoire with lower diversity at diagnosis had no significant impact on the outcome of these patients in our cohort.

**Conclusions:** In conclusion, we present the largest cohort of pediatric patients with HABMF analyzed by next-generation sequencing and flow cytometry so far. Even though our results show significantly increased activation of CD8pos T cells and presence of expanded clones, we did not confirm that these factors would be useful and significant for the prediction of treatment outcome. Large number of our patients does not fully respond to IST and require HSCT. Supported by AZV NV18-07-00430, NU20J-07-00028 a GAUK 534120

## 19. The role of the telomere length measurement in children and adolescents with aplastic anemia enrolled in the EWOG-SAA study

### A. Yoshimi^1^, M.Wlodarski^1,2^, D. Lebrecht^1^, A. Breier^1^, K. Kállay^3^, O Smith^4^, F. Locatelli^5^, J Buechner^6^, I. Bodova^7^, J. Sevilla^8^, M. Schmugge^9^, M. Bierings^10^, T. Masmas^11^, M. Dworzak^12^, V. Labarque^13^, J. Starý^14^, M. Matysiak^15^, K. Jahnukainen^16^, S. Polychronopoulou^17^, H. Tamary^18^, P. Kjollerstrom^19^, M. Kavcic^20^, M. Erlacher^1^, P. Noellke^1^, C. Niemeyer^1^, B.Strahm^1^

#### ^1^Department of Pediatrics and Adolescent Medicine, Division of Pediatric Hematology and Oncology, Medical Center, Faculty of Medicine, University of Freiburg, Freiburg, Germany; ^2^Department of Hematology, St. Jude Children’s Research Hospital, Memphis, USA; ^3^Dept. of Pediatric Hematology and Stem Cell Transplantation, Central Hospital of Southern Pest - National Institute of Hematology and Infectious Diseases, Budapest, Hungary; ^4^Pediatric Haematology, Our Lady’s Children’s Hospital, Dublin, Ireland; ^5^Department of Pediatric Hematology and Oncology, IRCCS Ospedale Pediatrico Bambino Gesù; Sapienza University of Rome, Rome, Italy; ^6^Department of Pediatric Hematology and Oncology, Oslo University Hospital , Oslo, Norway; ^7^Bone marrow transplantation unit, Detská fakultná nemocnica s poliklinikou v Bratislave, Bratislava, Slovakia; ^8^Hospital Infantil Universitario Niño Jesús, Madrid, Spain; ^9^Department of Hematology and Oncology, University Children’s Hospital, Zurich, Switzerland; ^10^Princess Máxima Center for Pediatric Oncology, Utrecht, The Netherlands; ^11^Department of Hematology, Center for Hemoglobinopathies, Herlev University Hospital, Herlev, Denmark; ^12^Department of Pediatrics, St. Anna Children’s Hospital and Children’s Cancer Research Institute (CCRI), Medical University of Vienna, Vienna, Austria; ^13^Department of Pediatric Hematology and Oncology, University Hospital Leuven Gasthuisberg, Leuven, Belgium; ^14^Department of Paediatric Haematology and Oncology, Second Faculty of Medicine, Charles University and University Hospital Motol, Prague, Czech Republic; ^15^Department of Paediatric Haematology and Oncology, Warsaw Medical University,, Warsaw, Poland; ^16^Division of Hematology-Oncology and SCT Children’s Hospital, University of Helsinki and Helsinki University Hospital, Hus, Finland; ^17^Department of Pediatric Hematology Oncology, Aghia Sophia Children’s Hospital, Athens, Greece; ^18^Hematology-Oncology Department, Schneider Children’s Medical Center of Israel, Petah Tiqva, Israel; ^19^Pediatric Hematology Unit, Hospital Dona Estefânia, Centro Hospitalar Universitário de Lisboa Central, Lisbon, Portugal; ^20^Unit of Oncology and Haematology, University Children’s Hospital, Ljubljana University Medical Centre, Ljubljana, Slovenia

**Introduction/ Aims:** Although telomere length (TL) analysis is useful to support the diagnosis of dyskeratosis congenita (DC), previous reports have shown that some patients with aplastic anemia (AA) and other bone marrow failure syndromes have short TL as well. The aims of this study were to evaluate the role of TL analysis in AA by comparing TL in AA patients with healthy controls and DC patients, evaluating the incidence of short TL in AA patients and analyzing the correlation between TL and outcomes after immunosuppressive therapy (IST).

**Methods:** Among 293 AA patients registered in the EWOG-SAA-2010 study between 2011 and 2020, TL in DNA extracted from granulocytes in bone marrow (BM, n=153) or peripheral blood (PB, n=40) was measured in 193 subjects using quantitative real-time PCR (adapted from RM Cawthon 2009). The results were compared with healthy pediatric controls (n=148) and DC patients (n=47). During the study period, 150 patients (M/F=92/58, med. age 10, range 1-18 years) received IST with anti-thymocyte globulin (ATGAM; n=110, thymoglobulin; n=40) and cyclosporine. The disease severity was very severe AA (VSAA), SAA and non-SAA (NSAA) in 127 (85%), 19 (13%) and 4 patients (3%), respectively. Variables associated with IST response were evaluated.

**Results:** The median TL in BM granulocytes was significantly shorter in AA patients than in healthy controls: 1.10 (range 0.21-5.22, n=153) vs 1.24 (range 0.78-2.75, n=76, p<0.01), but significantly longer than that of DC patients (0.55, range 0.30-0.81, n=26, p<0.01). There was no significant difference in TL in PB granulocytes between AA group and healthy control (median 1.09, range 0.23-1.97, n=40 vs 1.16, range 0.59-2.97, n=72, p=n.s.) but TL in PB granulocytes was significantly shorter in DC patients (0.41, range 0.26-1.08, n=21, p<0.01). Short TL (<1.P) was noted in 22/153 BM samples (15%) and 2/40 PB samples (5%) in AA patients, but none of them had DC phenotype. In 14 of these patients, mutations in most common genes (*DKC1*, *TINF2*, *RTEL1, TERT* and *TERC*) were excluded (no data in 10 patients). One-hundred-fifty AA patients were treated with IST; 55 patients (37%) responded to IST at day 180, 59 (n=39%) had no response, 33 patients (22%) received early HSCT, 2 patient received second IST and one patient died early. The 5-year overall and failure-free survivals after IST were 92% and 27%, respectively (failures defined by no response, relapse, clonal disease, secondary therapy and death). Significant short TL (<1.P) was observed in 15 of 110 evaluated patients (14%) and only one of them responded to IST. In univariate analysis, low absolute neutrophil count (ANC), short TL and thymoglobulin were associated with poor response. In multivariate analysis, low ANC was the most significant risk factor for poor response (p=0.015), followed by long interval between diagnosis and IST (p=0.037), while impact of short TL on response was statistically not significant (p=0.08) probably due to a small number of patients with short TL.

**Conclusions:** Although TL in BM granulocytes of AA patients was significantly shorter compared to healthy controls, clinical and genetic data did not indicate a high suspicion for unrecognized DC. However, further comprehensive genetic analysis is necessary to rule out mutations in any of the known or novel genes associated with DC. Furthermore, patients with short TL had a poor response to IST possibly indicating that short TL reflects a reduced stem cell reserve. These results might be used for improved therapy stratification in pediatric AA

## 20. Increased Age-related B cells in patients with Acquired Aplastic Anemia

### E.E Solomou, C. Salamaliki, N. Giannakoulas, P. Diamantopoulos, M. Palasopoulou, A. Galanopoulos, A.-N. Vyniou, G. Vassilopoulos, A. Symeonidis, A. Kattamis

#### University of Patras Medical School, Department of Internal Medicine, Patras, Greece

**Introduction:** Age-related B cells (ABCs) represent a small subpopulation of B cells. These cells express high levels of CD11c and CD19, they are CD21 negative and express T-bet in the nucleus. These T-bet+ ABCs are found increased in patients with autoimmune diseases (i.e. systemic lupus erythematosus, rheumatoid arthritis, and multiple sclerosis). Stimulation of B cells with antigens, Toll-like receptors and IFN-g leads to the formation of ABCs, which in turn “talk” to the T cells and stimulate them. Stimulation of T cells leads to IFN-g production, and this IFN-g may lead to further induction of T-bet expressing B cells (ABCs).

Aplastic anemia is a rare disease characterized by immune dysregulation. T cells in aplastic anemia are characterized by various intrinsic defects leading to increased IFN-g levels and Fas-mediated apoptosis of hematopoietic stem cells. We and others, have previously shown that the transcription factor.

**Aim**: In this study we wanted to examine the expression of ABCs in patients with aplastic anemia. Severity of aplastic anemia was defined based on standard criteria. We isolated peripheral blood mononuclear cells (PBMCs) from patients with aplastic anemia (n=11, age 8-50 years, 5 patients were children/adolescent) and five healthy, age- matched controls. Written informed consent was obtained from all study subjects. Cells were stained with the surface markers CD11c, CD19 and CD21, and subsequently analyzed using flow cytometry.

**Fig. 1 (abstract 20).**
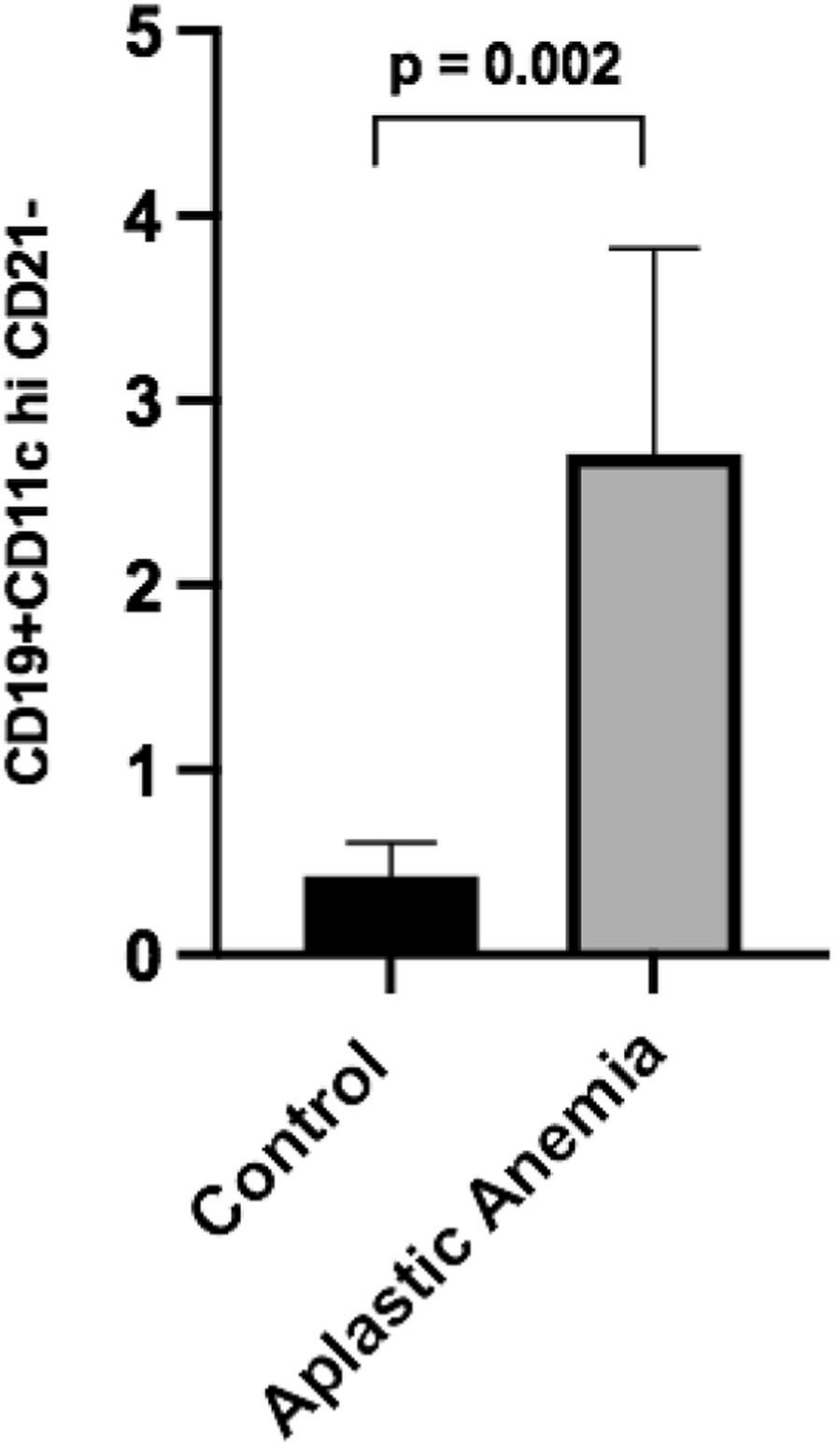
See text for description

**Methods/Results**: Patients with aplastic anemia at presentation showed increased numbers of circulating ABCs compared to healthy controls (2.71 ± 0.39% vs 0.43 ±0,09% respectively, p=0.002).(Fig. 1). Although the sample of patients is small to reach definitive conclusions, the number of circulating ABCs was directly correlated with aplastic anemia severity.

**Conclusions:** Our preliminary results suggest that aplastic anemia patients show expanded ABCs compared to age-matched control subjects, and these numbers are related to disease status. Further analysis of a larger pool of subjects is underway along with the examination of the specific transcription factor Bcl-6, that is implicated in T-bet expression in ABCs. These results will reveal the role of ABCs in the immune pathogenesis of aplastic anemia.

## 23. Clinical diagnostic value of telomere length measurement in inherited bone marrow failure syndromes

### A.Narita, S. Miwata, Y. Okuno, M. Hamada, M. Imaya, A. Yamamori, M. Wakamatsu, S. Kataoka, R. Taniguchi, N. Kawashima, E. Nishikawa, N. Nishio, S. Kojima, H Muramatsu, Y.Takahashi

#### Department of Pediatrics, Nagoya University Graduate School of Medicine, Nagoya, Japan

##### **Correspondence:** A.Narita

**Introduction:** Inherited bone marrow failure syndromes (IBMFS) occur in approximately 5%–30% of patients with bone marrow failure (BMF). Several investigators have demonstrated that telomere length (TL) is excessively short in patients with idiopathic aplastic anemia (AA) and non-DC IBMFS, including FA, SDS, and DBA. To assess the diagnostic value of TL, we measured TL in 133 patients with BMF and compared it in patients with DC, non-DC IBMFS, and AA.

**Methods:** We retrospectively studied 133 patients (68 male and 65 female) with BMF in Japan between 2013 and 2018. We collected peripheral blood samples at diagnosis from all patients, measured TL from peripheral blood lymphocytes, and performed targeted sequencing analysis covering 184 genes associated with IBMFS. We divided 133 patients into three groups: DC, non-DC IBMFS, and AA.

**Results:** Using targeted sequencing, we detected from 24 patients (18%) 31 pathogenic variants (3 nonsense, 14 missense, 5 frameshift, 4 splice site, and 4 deletion) of known causative IBMFS genes, including *TINF2* (n = 6), *TERT* (n = 3), *FANCA* (n = 6), *FANCG* (n = 3), *RPL5* (n = 2), *RPS19* (n = 1), *RPS17* (n = 1), *SBDS* (n = 1), and *BLM* (n = 1). Homozygous mutations were found in 3 patients (2 in *FANCC* and 1 in *FANCA*), compound heterozygous in 4 patients (2 in *FANCA* and 1 each in *FANCG* and *SBDS*), hemizygous in 3 patients in *FANCA*, and heterozygous in 14 patients (6 in *TINF2*, 3 in *TERT*, 2 in *RPL5*, and 1 each in *RPS17, RPS19,* and *BLM*). Out of the 133 patients, 11 were diagnosed with DC (8%), 15 with non-DC IBMFS (11%), and 107 with AA (81%). Of the 11 patients with DC, 9 were genetically diagnosed (6 in *TINF2* and 3 in *TERT*), and those without diagnostic genetic mutations were diagnosed following clinical diagnostic criteria. The 15 non-DC IBMFS cases consisted of 9 FA, 4 DBA, 1 SDS, and 1 Bloom syndrome. Physical anomalies were observed in 11 of 15 (73%) patients. We compared the clinical characteristics of patients with DC, non-DC IBMFS, and AA. Median TL in the patients with DC, non-DC IBMFS, and AA were −3.50 SD (range, −5.73 to +0.83 SD), −1.89 SD (range, −4.74 to +2.05 SD), and −0.84 SD (range, −4.27 to +4.00 SD), respectively. Patients with DC showed significantly shorter TL compared with those with non-DC IBMFS (P = 0.031) and AA (P < 0.001). Furthermore, patients with non-DC IBMFS tended to show shorter TL than those with AA (P = 0.096). To validate the efficacy of TL measurement in diagnosing DC and IBMFS, receiver operating characteristic curves identified two cut-off values with the optimum sensitivity and false positive rate (1-specificity) combination, <−2.19 SD and <−1.71 SD, defined as “very short TL” and “relatively short TL,” respectively. For the diagnosis of patients with IBMFS, the TL cut-off value at −1.71 SD (relatively short TL) yielded relatively high negative (0.921; 95% confidence interval [CI], 0.873–0.958) and moderately positive predictive values (0.432 [95% CI; 0.333–0.505]). Of the total cohort, 44 patients (33%) were classified with “relatively short TL”, which was significantly more frequent (P < 0.001) in DC (10/11, 91%) and non-DC IBMFS (9/15, 60%) than in AA (25/107, 23%).

**Conclusions:** This study confirms that a relatively short TL was found in a significant proportion of patients with DC and non-DC IBMFS, indicating the clinical diagnostic value of TL measurement in identifying patients who need further testing, particularly comprehensive genetic analysis.

## 24. Gain-of-Function Mutations in Replication Protein A1 (RPA1) Identified in Individuals with Telomere Biology Disorders

### R. Sharma^1^, S. Sahoo^1^, S. Polychronopoulou^2^, M. Honda^3^, C. Goodings^1^, C.Kelaidi^2^, P. Revy^4^, C. Niemeyer^5^, M.Erlacher^5^, M. Wlodarski^1,5^

#### ^1^St Jude Children’s Research Hospital, United States; ^2^Aghia Sophia Children’s Hospital, Athens, Greece; ^3^University of Iowa, Iowa City, IA, USA; ^4^Université de Paris, Imagine Institute, Paris, France; ^5^University of Freiburg, Freiburg, Germany

##### **Correspondence:** R. Sharma

**Introduction:** Human telomere biology disorders (TBD) are hereditary multisystemic disorders that are caused by premature telomere shortening due to loss-of-function mutations in telomere-associated genes, however many patients remain without a genetic resolve. Here, we characterize germline heterozygous missense variants in *RPA1* identified in four unrelated probands presenting with varying phenotypes of TBD. RPA1 is an essential single-strand DNA-binding protein involved in DNA replication and repair.

**Aim:** The aims of our study were to delineate the clinical phenotype of individuals with *RPA1* variants, characterize the genetic landscape and mechanisms of somatic genetic rescue, and asses if *RPA1* germline mutations are deleterious in biochemical and cellular systems.

**Methods:** All patients underwent detailed evaluation and had negative workup for inherited bone marrow failure (BMF) syndromes and TBD associated genes. Genomic studies (whole exome sequencing, ultradeep sequencing, single cell multi-omics, RNAseq, ddPCR-based haplotype phasing), telomere length assessment, biochemical assays (DNA and telomere binding studies, telomerase assay), and *in vitro* modeling using induced pluripotent stem cells (iPSC) were performed.

**Results**: We found four unrelated TBD patients to carry three distinct (two confirmed *de novo*) missense heterozygous mutations in *RPA1*. All three mutations cluster to the DNA binding domain A (DBD-A), which has previously been shown to be the central functional domain involved in binding to single stranded DNA (ssDNA). The patients had heterogenous clinical manifestations unified by short telomeres and TBD features, including BMF, myelodysplastic syndrome, lymphopenia, pulmonary fibrosis, and mucocutaneous triad. Using forester resonance energy transfer (FRET) DNA binding assay, we show increased binding of germline *RPA1* mutants to ssDNA and telomeric DNA, consistent with a gain-of-function effect. To understand the effect of *RPA1* variants on telomere length regulation and hematopoietic development, we used CRISPR/Cas9-mediated mutagenesis to introduce patient p.E240K mutation (denoted as *RPA1*^E240K^) in a healthy donor iPSC line (*RPA1*^WT^). RPA1^E240K^ iPSC model exhibited premature telomere shortening at iPSC and hematopoietic progenitor level, as well as reduced hematopoietic differentiation. Finally, we identified somatic rescue events in hematopoiesis of patient with p.E240K mutation, which correlated with long term stable clinical phenotype. These included a somatic *RPA1* p.K579* mutation (in cis with p.E240K) causing RNA degradation, and an independent clone with uniparental isodisomy 17p resulting in duplication of wild type *RPA1* allele.

**Conclusions**: We identified *RPA1* mutations to be associated with telomere shortening in humans, which calls for careful consideration of *RPA1* missense variants in the workup of patients with TBD phenotypes. The mutations are likely gain-of-function with increased DNA binding capacity, and negative selective pressure resulting in somatic rescue in hematopoiesis. We speculate that germline *RPA1* alterations may be more common in human disease, given that somatic *RPA1* mutations occur in ~1% of cancers. Additional efforts are needed to not only find further pathogenic *RPA1* mutations but to also elucidate the role of *RPA1* in human telomere biology.

## 25. Strategies of NGS data analysis to maximize efficiency of identification of causative gene defects in non-malignant hematological diseases and inherited bone marrow failure syndromes

### J. Berner^1,2,3^, M. Haimel^1,3,4^, D. Mayr^1,2,3^, W. Novak^2^, R. J. Heredia^1,3,4,5^, A. Frohne^1,3,4^, V. Hertlein^1,3,4^, M. Dworzak^1,2^, L. Kager^2^, K. Boztug^1,2,3,4,5^

#### ^1^St. Anna Children’s Cancer Research Institute (CCRI), Vienna, Austria; ^2^St. Anna Children’s Hospital, Department of Pediatrics and Adolescent Medicine, Medical University of Vienna, Vienna, Austria; ^3^Ludwig Boltzmann Institute for Rare and Undiagnosed Diseases, Vienna, Austria; ^4^CeMM Research Center for Molecular Medicine of the Austrian Academy of Sciences, Vienna, Austria; ^5^Department of Pediatrics and Adolescent Medicine, Medical University of Vienna, Vienna, Austria

**Introduction:** Next-generation sequencing (NGS) technology has revolutionized the genetic diagnosis of rare diseases, and variants in an increasing number of genes are known to cause non-malignant hematologic disorders. Defining a workflow for reliable and sensitive genetic diagnoses in these patients is essential to optimize patient management and treatment. In many cases, genetic diagnoses can be made with predefined sequencing panels. Other patients require whole-exome (WES) or whole-genome sequencing to define the causative genetic variant. However, for a significant proportion of patients, a clear genetic etiology cannot be identified. This may be due to a non-monogenic etiology but is also accounted for by limitations in the identification and interpretation of candidate pathogenic variants, including incomplete knowledge of gene- disease associations or technical shortcomings. As both the knowledge pool of molecular disease causes and bioinformatic means are rapidly advancing, increasing numbers of previously unsolved cases can be solved. Thus, it is imperative to revise previously unsolved sequencing data rigorously and constantly. Within the European Ribosomopathy Consortium (RiboEurope), we have conceptualized the strategic reanalysis of NGS data to identify the genetic etiology in previously unsolved cases.

**Aims:**To define and optimize a genetic diagnostic strategy for rare non-malignant hematologic diseases.

**Methods:** 189 patients were analyzed using comprehensive panel sequencing (variable panels of 300-600 genes; 119 patients) or a more recently implemented pipeline based on WES followed by the targeted analysis of a constantly updated virtual gene panel (currently including more than 600 genes; 70 patients). In addition to SNPs and small indels, the workflow included copy number variant calling, deep-learning-based splice variant prediction, HPO terms and integration of publicly available databases (gnomAD). Candidate variants meeting basic criteria compatible with pathogenicity, such as a plausible allele frequency and effect on the gene product, were individually evaluated using literature, mouse phenotype data, prediction tools, and expert opinion. If, in the WES-based approach, no candidates were identified, the analysis was extended to include exome-wide data. Regular updates and additions to the individual tools, annotations, and the virtual panel as well as the subsequent reanalysis of unsolved cases were an integral part of the pipeline.

**Results:** We have successfully implemented a comprehensive, ever-evolving workflow for the diagnosis of patients with non-malignant hematologic diseases, integrating state-of-the-art NGS technology, bioinformatics tools, constantly updated clinical and molecular data, and expert opinion. The total solve rate in the cohort amounted to 40%, and was increased among patients analyzed with the more recently implemented WES-based pipeline, which allows a higher flexibility in the data analysis and reanalysis (43% as compared to 39% in physical panels). Due to the ongoing constant revision of the cohort using updated tools and reference information, the solve rate is expected to further increase in the future.

**Conclusions:** With this study, we present a comprehensive pipeline that increases the diagnostic capacities of NGS data for monogenic non-malignant hematologic disorders.

## 26. A human model of clonal evolution in Fanconi anemia

### W. Marion^1^, O. Aumais^1^, D. Wang^1^, C.-C. Chou^2^, P. Sensharma^1^, S. Boettcher^3^, S. Ruiz-Torres^4^, S.I. Wells^4^, A. Shimamura^1^, E. Lummertz da Rocha^5^, B. L. Ebert^3^, R. Grant Rowe^1^

#### ^1^Boston Children’s Hospital, Boston MA, USA; ^2^Massachusetts Institute of Technology, Cambridge, MA, USA; ^3^Dana-Farber Cancer Institute, Boston, MA, USA; ^4^Cincinnati Children’s Hospital, Cincinnati, OH, USA; ^5^Federal University of Santa Catarina, Florianopolis, Brazil

**Introduction:** Many forms of pediatric bone marrow failure (BMF) bear a predisposition to leukemia. Patients with Fanconi anemia (FA) are at high risk of developing myeloid malignancy. This typically occurs in the setting of BMF, where somatic mutations drive quiescent hematopoietic stem cell (HSC) clones to undergo aberrant self-renewal with impaired differentiation, manifesting as myelodysplastic syndrome (MDS). Additional somatic mutations result in further dysregulation of self-renewal resulting in frank acute myeloid leukemia (AML), a significant source of morbidity and mortality in FA. This process of clonal evolution is a difficult clinical challenge in FA given the sensitivity of FA patients to genotoxic chemotherapy agents required to treat MDS and AML. Therefore, improved understanding of the process of clonal evolution of failing HSCs toward pre-leukemic and leukemic stem cells would benefit patients with FA and BMF more broadly through the derivation of improved surveillance strategies to guide the use of curative allogeneic HSC transplantation as well as the delineation of novel molecular vulnerabilities in FA-associated MDS and AML.

**Aims:** To understand clonal evolution in FA in a human disease model.

**Methods:** In this study, we used gene editing of human induced pluripotent stem cells from FA patients to gain understanding of clonal evolution in FA. We performed directed differentiation of these iPSCs to hematopoietic progenitor cells for analysis of DNA repair and blood stem cell properties.

**Results:** We find that somatic mutations commonly occurring in MDS and AML confer quiescent FA hematopoietic progenitors with aberrant self-renewal and that the incorporation of an activating oncogene mutation can trigger leukemogenesis. Gene expression analysis showed that FA MDS and AML cells express ectopic stem cell transcriptional signatures and lose signatures of inflammation and terminal differentiation typical of FA hematopoietic progenitors. Comparative functional analysis of human FA progenitors, MDS-like cells, and leukemia cells revealed that cell cycle checkpoints hyperactivated in progenitors were blunted in MDS and AML cells as a result of somatic mutations, and that AML cells continue to show genomic instability and accumulation of DNA damage despite ongoing proliferation.

**Conclusions:** Overall, these findings provide new insight into clonal evolution of FA hematopoietic progenitors to MDS and AML in a renewable human system and provide a platform for therapeutic discovery in myeloid neoplasms associated with FA. Our work provides a human based model system that can be adapted to the study of clonal evolution and leukemogenesis in many forms of pediatric BMF.

## 27. A Novel Classification of Hematologic Conditions in Patients with Fanconi Anemia – A Report from the German FA Registry

### Y.L. Behrens^1†^, G. Göhring^1†^, R. Bawadi^1^, S. Cöktü,^2^ C. Reimer^2^, B. Hoffmann^2^, B. Sänger^2^, S. Käfer^1^, F. Thol^3^, M. Erlacher^4^, C.M. Niemeyer^4^, I. Baumann^5^, R. Kalb^6^, D. Schindler^6^, C.P. Kratz^2^

#### ^1^Department of Human Genetics, Hannover Medical School, Carl-Neuberg-Str.1, 30625 Hannover, Germany; ^2^Pediatric Hematology and Oncology, Hannover Medical School, Hannover, Germany; ^3^Department of Hematology, Hemostasis, Oncology and Stem Cell Transplantation, Hannover Medical School, 30625 Hannover, Germany; ^4^Division of Pediatric Hematology and Oncology, Department of Pediatrics and Adolescent Medicine, University Medical Center Freiburg, Faculty of Medicine, University of Freiburg, Germany and German Cancer Consortium (DKTK), Freiburg, Germany and German Cancer Research Center (DKFZ), Heidelberg, Germany; ^5^Institute of Pathology, Kaufbeuren, Germany; ^6^Department of Human Genetics, University of Würzburg, Biocenter, Würzburg, Germany

##### **Correspondence:** C.P. Kratz

†These authors contributed equally to this work.

**Introduction:** Fanconi anemia (FA) is an inherited bone marrow failure syndrome associated with an increased risk of myelodysplastic syndrome (MDS) and acute myeloid leukemia (AML). The bone marrow in patients with FA is typically hypocellular and can mimic other conditions such as acquired severe aplastic anemia. The presence of cytopenia and myelodysplastic features alone are not sufficient for the diagnosis of MDS in FA – with the exception of distinct subtypes such as refractory anemia with ring sideroblasts or in cases with rapid rise in cellularity in whom MDS needs to be considered. In patients with FA, the diagnosis of MDS requires an elevated blast count and/or other definite signs of transformation, such as duplication of chromosome 3q, deletion 7q, or monosomy 7. Until now, however, an internationally accepted classification of myeloid neoplasia in patients with FA is lacking.

**Methods:** We studied 86 patients enrolled in the German FA registry and performed an algorithm for the diagnosis of benign and malignant hematologic conditions in FA that is based on blast percentage and cytogenetics. In addition to FA-BMF, FA-MDS-without excess blasts (FA-MDS-non EB; in the young generally with a pattern of refractory anemia of childhood), FA-MDS with excess blasts (FA-MDS-EB) and FA-AML, the classification includes the new category FA with cytogenetic aberration of indeterminate potential (FA-AIP). The new classification FA-AIP is characterized by cytogenetic aberrations such as duplication of chromosome 1q, deletion 6p, or deletion 7p that remain stable over a prolonged period without transformation. The proposed diagnostic algorithm avoids the misdiagnosis of myeloid neoplasia and averts the immediate indication for HSCT. In addition, the new algorithm serves as a model for the classification of myeloid neoplasms in patients with a germline predisposition. The concept of AIP may also apply to other inherited bone marrow failure syndromes such as Shwachman-Diamond syndrome.

## 28. *DNMT3A* and activating *TP53* germline mutations mimicking Diamond-Blackfan anemia

### G. Ovsyannikova, D. Fedorova, A. Pavlova, T. Konyukhova, M. Maschan, N.Smetanina

#### Dmitry Rogachev National Medical Research Center of Pediatric Hematology, Oncology and Immunology, Moscow, Russian Federation

**Keywords:** hereditary predisposition to MDS/AML*; DNMT3A* germline mutation; Tatton–Brown–Rahman syndrome; activating *TP53* germline mutation.

**Introduction:** The genetic diagnosis of bone marrow failure disorders in childhood is especially important since the disorder may constitute a hereditary predisposition syndrome to MDS/AML. Here we describe the clinical course of 3 children diagnosed with hypoplastic anemia who were later found to have germline mutations in *TP53* or *DNMT3A*.

**Methods**: All patient samples had been obtained with informed consent, and the study had been approved by the local ethics committee. Genetic testing was done by Sanger sequencing for single gene analysis or target next-generation sequencing (NGS) on the MiSeq/NextSeq (Illumina, USA) using Bone Marrow Failure Syndrome custom gene panel.

**Results**: All 3 pts had profound macrocytic hypoproliferative anemia diagnosed at birth (patient 1), at 2 months (patient 2) or at 7 months (patient 3) requiring transfusions of packed red blood cells (RBCs). Bone marrow (BM) investigation at the first evaluation revealed normocellular marrow with no increase in blasts and red cell hypoplasia. The primary investigation ruled out the most common inherited bone marrow failure syndromes. Considering the age of manifestation, a preliminary diagnosis of Diamond-Blackfan anemia (DBA) was suspected. With NGS, patient 1 was found to have *DNMT3* germline mutation p.(Arg882His), which is a hotspot mutation in the catalytic domain and linked to Tatton–Brown–Rahman syndrome.

Besides anemia, a mild thrombocytopenia (57-108 X 10^9^ L) was revealed at the age of 9 months. At age 12 months, he was started on prednisolone and his hemoglobin (Hb) level normalized. Prednisolone therapy was discontinued at age 6.7 years, and at age 11 years, Hb level started to decline again (7.3 g/dl). The BM aspirate showed a hypocellular marrow without an increase in blasts, marked myeloid dysplasia and a decrease in erythroid precursors and megakaryocytes. Bone marrow karyotype was normal. At age 13 years, patient had a disease progression in the form of profound neutropenia, high transfusion dependence of RBCs and platelets, and a reduction in number of B- and T-cells. Hematopoietic stem cell transplantation is planned. The non-hematological phenotype of patient 1 included mild dysmorphic facial features, excessive weight gain and febrile seizures. In patients 2 and 3, activating *TP53* mutations were found: p.Ser362Alafs*8 and c.1100+1G>C, which cause truncation of the protein resulting in the loss of 32 residues from the C-terminal domain. For the diagnosis DBA both patients were started on L-leucine at the age of 2 years. They became transfusion independent and at the time of writing continue to do so 2.5 and 7 years after starting L-Leucine therapy. The non-hematological phenotype of both cases included mild microcephaly and short stature; in patient 2, cardiac anomalies and hepatic cavernous hemangioma with portal hypertension and moderate splenomegaly were also observed.

**Conclusion:** The continuing effort to analyze clinical and genetic data on patients with unresolved bone marrow failure disorder is of great especially importance. It is the prerequisite for personalized surveillance and counseling of patients and carriers

## 30. Outcomes of second hematopoietic stem cell transplantation In patients with relapsed juvenile myelomonocytic leukemia

### L.Vinci^1^, C. Flotho^1^, P. Noellke^1^, M.Erlacher^1^, D. Lebrecht^1^, R. Masetti^2^, V. de Haas^3^, B. De Moerloose^4^, M. Dworzak^5^, H. Hasle^6^, M.Schmugge^7^, J. Starý^8^, D. Turkiewicz^9^, M. Ussowicz^10^, A. Catala^11^, J. Buechner^12^, K. Jahnukainen^13^, K. Kállay^14^, O. Fabri^15^, O. Smith^16^, G. Göhring^17^, F. Locatelli^18^, B. Strahm^1^, C.M. Niemeyer^1^, A. Yoshimi^1^

#### ^1^Department of Pediatrics and Adolescent Medicine, Division of Pediatric Hematology and Oncology, Medical Center, Faculty of Medicine, University of Freiburg, Freiburg, Germany; ^2^Department of Pediatric Oncology and Hematology, University of Bologna, Bologna, Italy; ^3^Princess Maxima Center, Diagnostic Laboratory / DCOG Laboratory, Utrecht, The Netherlands; ^4^Department of Pediatric Hematology-Oncology and Stem Cell Transplantation, Ghent University Hospital, Ghent, Belgium; ^5^Department of Pediatrics, St. Anna Children’s Hospital, Medical University of Vienna, Vienna, Austria; ^6^Department of Pediatrics, Aarhus University Hospital Skejby, Aarhus, Denmark; ^7^Department of Hematology and Oncology, University Children’s Hospital, Zurich, Switzerland; ^8^Department of Pediatric Hematology and Oncology, Charles University and University Hospital Motol, Prague, Czech Republic; ^9^Department of Pediatric Oncology/Hematology, Skåne University Hospital, Lund, Sweden; ^10^Department of Pediatric Hematology and Oncology, BMT Unit CIC 817, Wroclaw Medical University, Wroclaw, Poland; ^11^Department of Hematology and Oncology, Hospital Sant Joan de Deu, Barcelona, Spain; ^12^Department of Pediatric Hematology and Oncology, Oslo University Hospital, Oslo, Norway; ^13^Division of Hematology-Oncology and SCT Children’s Hospital, University of Helsinki and Helsinki University Hospital, Hus, Finland; ^14^Department of Pediatric Hematology and Stem Cell Transplantation, Central Hospital of Southern Pest - National Institute of Hematology and Infectious Diseases, Budapest, Hungary; ^15^Department. of Haematology and Transfusiology, National Institute of Children’s Diseases Faculty of Medicine, Comenius University, Bratislava, Slovakia; ^16^Pediatric Haematology, Our Lady’s Children’s Hospital, Dublin, Ireland; ^17^Institute of Cell and Molecular Pathology, Hannover Medical School, Hannover, Germany; ^18^Department of Pediatric Hematology and Oncology, Bambino Gesu Children’s Hospital, Sapienza, University of Rome, Rome, Italy

##### **Correspondence:** L.Vinci

**Introduction/Aims:** Allogeneic hematopoietic stem cell transplantation (HSCT) is the only curative therapy for most patients with juvenile myelomonocytic leukemia (JMML). Relapse remains the major cause of treatment failure after HSCT, and therapy options for relapse are limited. The aim of this study is to analyze the outcome of JMML patients who received a second HSCT for relapse after the first grafting procedure.

**Methods:** Seventy-eight patients registered in the EWOG-MDS studies received a second HSCT for relapse following a first HSCT performed between 07/1988 and 01/2020. After excluding 10 patients with insufficient data, 68 patients were included in the analysis. The median age at diagnosis was 3.0 years (0.2 – 15.1) and at second HSCT 4.6 (1.0 - 15.8) years. The intervals between first HSCT and relapse and between first and second HSCTs were 247 (15 - 788) days and 352 (35 - 907) days, respectively. A *PTPN11* driver mutation was known in 42 patients (61%). Most patents (n=49) did not receive any anti-leukemic therapy prior to the second allograft, while 10, 9, 13 and 2 patients were treated with low-dose chemotherapy, azacitidine, donor-leukocyte infusion (DLI) and splenectomy, respectively. Only one patient (treated with azacitidine) was in clinical remission before the second HSCT. The majority of patients (n=54) had received a busulfan/cyclophosphamide/melphalan (Mel) regimen for first HSCT (others n=14). For second HSCT a total body irradiation based (n=28), treosulfan/fludarabine (Flu)/thiotepa (TT) (n=14) and Mel/Flu/TT (n=9) regimens were applied (others n=9). For second HSCT, 16 patients were transplanted from an HLA-matched sibling donor (MSD), 9 patients from a haplo-identical family donor and 43 patients from an unrelated donor (UD). The same donor was used for both first and second HSCTs in 31 children. Stem cell source was bone marrow, peripheral blood and cord blood in 37, 26, and 6 patients, respectively.

**Results***:* Engraftment was achieved in 61 of 68 patients, 1 patient encountered a secondary graft failure. The incidence of acute II-IV/III-IV and chronic graft-versus-host disease (GVHD) were 52% / 22% and 37%, respectively. With a median follow-up of 7.7 years, the 5-year-overall survival, disease free survival, cumulative incidences of relapse and non-relapse mortality after second HSCT were 40%, 36%, 41% and 23%, respectively. Patients with older age at second HSCT (> 3 years) and early relapse (< 180 days) after first HSCT tended to have a worse outcome, while mutational subtype, type of donor, change of donor for second HSCT, and conditioning regimen did not affect outcome. Relapse after the second HSCT was the main cause of death (n = 23), while 17 children succumbed due to transplantation-related causes; graft failure (n = 4), acute and chronic GVHD (n = 3 and 4), early transplant toxicity with multi-organ failure (n = 1), infections (n= 4) and chronic lung disease (n = 1).

**Conclusions:** Relapsed JMML following HSCT can successfully be treated with a second allograft in about a third of the patients. However, in our experiences only about half of the patients who relapsed after first HSCT receive a second allograft. Further efforts to reduce relapses after first HSCT and development of novel therapies for relapse are necessary

## 31. JMML treatment strategies prior to allogeneic stem cell transplantation and survival outcomes – a single-centre experience

### M. Balasch-Carulla^1,2^, A. Margarit-Soler^2^, G. Lucchini^2^, P. Patel^3^, J. Chalker^4^, A. Rao^1^

#### ^1^Department of Haematology, Great Ormond Street Hospital for Children, NHS Foundation Trust, London, United Kingdom; ^2^Great Ormond Street Hospital for Children, NHS Foundation Trust, London, United Kingdom; ^3^Department of Pharmacy, Great Ormond Street Hospital, NHS Foundation Trust, London, United Kingdom; ^4^UCL Great Ormond Street Institute of Child Health, London, United Kingdom

##### **Correspondence:** M. Balasch-Carulla

**Introduction:** Juvenile myelomonocytic leukaemia (JMML) is a unique myeloproliferative/myelodysplastic syndrome where HSCT is the only curative treatment for children with somatic RAS-MAPK pathway mutations and aggressive disease. Differential methylation patterns have led to the use of hypomethylating agents, to reduce disease burden prior to transplant. Achieving a molecular remission prior to transplant has been reported to prevent post-transplant relapse.

**Aim:** The aim of our study was to assess the response to treatment pre HSCT and the overall outcomes in our cohort of patients who received Azacytidine as part of their pre SCT treatment. We aimed to unify the response assessment based on the response criteria defined by Niemeyer et al (Niemeyer CM et al, Criteria for evaluating response and outcome in clinical trials for children with juvenile myelomonocytic leukemia. Haematolog ica. 2015 Jan;100(1):17-22. doi: 10.3324/haematol.2014.109892. PMID: 25552679; PMCID: PMC4281308)

**Methods:** From 1^st^ of January 2015 until 31^st^ of December 2020 we systematically reviewed treatment and outcomes of 17 patients with confirmed molecular diagnosis of JMML. They all received Azacytidine as part of their treatment. All continuous variables were presented as medians and ranges and categorical variables as counts and percentages using Fisher exact test for comparison.

**Results:** The 17 JMML children have been classified based on their mutational subgroups of PTPN11

n=4, KRAS n=6, NRAS n=4 and NF1 n=3. Monosomy 7 was present in 5 out 17 patients, with an over-representation in the KRAS cohort. Poor prognostic features of age > 4 yrs/raised HbF and low platelet count were present across all 4 groups. Azacitidine was recommended as the preferred treatment option for all newly diagnosed children with JMML, 6/17 (35%) children received AZA alone, 11/17 received AZA sequentially with other therapies. In 7/17 (41%) children their clinical progression dictated the need to escalate to more aggressive chemotherapy regimens like FLA (Fludarabine/Cytarabine). Only 9/17 (52%) received a minimum of 3 cycles of Azacitidine. The mean interval from diagnosis to transplant was 6 months. Based on the response criteria described by Niemeyer et al, we achieved complete clinical remission in 2 patients (12%), stable disease in 2 patients (12%), partial clinical remission in 7 patients (41%), progressive disease in 5 patients (29%) and 1 patient with overt relapse (6%). All the patients underwent SCT. Out of 17 patients, 15 are currently alive with an OS of 88% with a median follow up of 5 years. Four patients relapsed (2 cPR, 1 cSD and 1 cRel), the event free survival is 82% (death related-disease progression, relapse, sepsis in the context of severe GVHD).

**Conclusions:** Treatment options recommended prior to transplant in JMML are variable. All 17 patients inour cohort received Azacitidine. However only 52% of the cohort received a minimum of 3 cycles of therapy. Escalation to aggressive chemotherapy was required in 41% of the cohort. Irrespective of the treatment given, 65 % of the cohort achieved cCR, cSD and pCR. Relapses occurred across all mutational subgroups. Interestingly, the 4 patients who relapsed did not have progressive disease at the time of HSCT. We did not measure mutation specific VAF at the time of HSCT. Our data highlights the challenges to recommending a uniform treatment strategy prior to transplant in JMML in view of the variable clinical course in these patients. There is an urgent need to standardize biomarkers of disease response prior to HSCT, which predict for post-transplant relapse. In the future, novel therapies in JMML will need to address the variability of disease progression and assessed using validated prognostic biomarkers of disease relapse.

## 32. Using BH3-mimetics in combination with azacitidine to fight juvenile myelomonocytic leukemia

### Y. Wu^1^, S. Bohler^1^, N. Koleci^1^, L. Gallego-Villar^1^, G. Andrieux^2^, J. Rajak^1^, K. Aumann^3^, C. Niemeyer^1^, C. Flotho^1^, M. Erlacher^1^

#### ^1^University Medical Center Freiburg, Division of Pediatric Hematology and Oncology, Freiburg, Germany; ^2^University Medical Center Freiburg, Institute of Medical Bioinformatics und Systems Medicine IBSM, Freiburg, Germany; ^3^University Medical Center Freiburg, Institute of Pathology, Freiburg, Germany

**Introduction:** Juvenile myelomonocytic leukemia (JMML) is a highly aggressive and life-threatening myeloproliferative neoplasm of early childhood. It is driven by oncogenic RAS signaling in addition to epigenetic deregulation. The only curative treatment until now is allogeneic stem cell transplantation (HSCT), bearing risks like graft failure or relapse. The DNA methyl transferase inhibitor Azacitidine is effective against JMML but unfortunately not all patients respond to this treatment. Therefore, alternative therapeutic strategies are urgently required. BH3-mimetics directly inhibit anti-apoptotic BCL-2 proteins (i.e. BCL-2, BCL-XL, MCL-1 and/or BCL-W) thereby inducing apoptosis at the mitochondrial level. These compounds can either be used alone or in combination with other anticancer drugs to achieve synergistic effects. Venetoclax, a specific BCL-2 inhibitor, was FDA-approved in combination with hypomethylating agents for the treatment of acute myeloid leukemia (AML) in adults. Here, we addressed the question if different BH3-mimetics, alone or in combination with azacitidine, can be used to fight JMML.

**Methods/Results:** Our research questions were addressed with experiments done *in vivo* and *in vitro*, in a patient-derived xenograft (PDX) mouse model and in naïve JMML cells, respectively. PDX mice were treated with ABT737, an analogue of navitoclax known to inhibit BCL-2, BCL-XL and BCL-W. Treatment was initiated 8 weeks after xenotransplantation of splenic JMML cells. ABT737 was given for 28 days (75 mg/kg/d), and on day 29, mice were sacrificed and analyzed. One group of mice was treated with azacitidine as described earlier (i.e. 2 cycles à 5 days followed by 9-day breaks, 3 mg/kg/d). Both ABT737 and azacitidine led to efficient leukemia depletion but only azacitidine selectively depleted leukemia-initiating cells (LICs). Next, we analyzed whether both drugs act synergistically and could be used at lower concentration when given in combination, with the aim to minimize any potential side effect. Indeed, a 28 days long treatment with 0.75 mg/kg/d azacitidine (5 days on, 9 days off) and 50 mg/kg/d ABT737 (daily) did not only completely reduce leukemia burden but also depleted all LIC as confirmed by serial transplantation experiments. To dissect the roles of BCL-2 and BCL-XL, both inhibited by ABT737, and to additionally analyze the role of MCL-1 in the survival of JMML cells, we performed *in vitro* experiments using the specific inhibitors ABT199, A1155463 and S63845, respectively. When used alone, only ABT737 and the MCL-1 inhibitor S53845 killed JMML cells efficiently. However, synergistic effects were noted between azacitidine and the BCL-XL inhibitor A1155463. Azacitidine treatment resulted in the upregulation of pro-apoptotic BCL-2 proteins (i.e. BIK, PUMA, BAD and BMF) and a slight downregulation of MCL-1 indicating that azacitidine and the BCL-XL inhibitor synergized at the level of the BCL-2 protein family.

**Conclusions:** To conclude, our study indicates that combining azacitidine with BH3-mimetics could be a new and promising way to treat JMML patients, especially the ones that became resistant to azacitidine monotherapy.

## 33. NF1 Tumor Suppressor Gene Inactivation in Juvenile Myelomonocytic Leukemia: Genetic Evidence and Suggestions for Diagnostic Work-Up

### S. Ramamoorthy^1,2^, D. Lebrecht^1^, D. Schanze^3^, I. Schanze, MD^3^, I. Wieland^3^, M.H. Albert, ^4^, A. Borkhardt^5^, D. Bresters^6^, J. Büchner^7^, A. Catala^8^, V. De Haas^9^, M. Dworzak^10^, M. Erlacher^1,11^, H. Hasle,^12^, K. Jahnukainen^3^, F. Locatelli^14^, R. Masetti^15^, J. Stary^6^, D.Turkiewicz^17^, L Vinci^1^, M. Wlodarski^1^, A.Yoshimi^1^, M.E. Hess^2^, G. Andrieux^2,11^, M. Boerries^2,11^, C.M. Niemeyer^1,11^, Martin Zenker^3^ and Christian Flotho^1,11^

#### ^1^Division of Pediatric Hematology and Oncology, Department of Pediatrics and Adolescent Medicine, Medical Center, Faculty of Medicine, University of Freiburg, Freiburg, Germany; ^2^Institute of Medical Bioinformatics and Systems Medicine, Medical Center, Faculty of Medicine, University of Freiburg, Freiburg, Germany; ^3^Human Genetics, University of Magdeburg, Magdeburg, Germany; ^4^Department of Pediatric Hematology and Oncology, Dr. v. Hauner Children’s Hospital, University Hospital, LMU, Munich, Germany; ^5^Department of Pediatric Oncology, Hematology and Immunology, University of Dusseldorf, Dusseldorf, Germany; ^6^Princess Maxima Center for Pediatric Oncology, Utrecht, Netherlands; ^7^Department of Pediatric Hematology and Oncology, Oslo University Hospital, Oslo, Norway; ^8^Department of Hematology and Oncology, Hospital Sant Joan de Déu, Barcelona, Spain; ^9^Princess Maxima Center for Pediatric Oncology, Diagnostic Laboratory / DCOG Laboratory, Utrecht, Netherlands; ^10^St. Anna Children’s Hospital and Cancer Research Institute, Medical University of Vienna, Vienna, Austria; ^11^German Cancer Consortium (DKTK) and German Cancer Research Center (DKFZ), partner site Freiburg, Freiburg, Germany; ^12^Department of Pediatrics, Aarhus University Hospital Skejby, Aarhus, Denmark; ^13^Division of Hematology-Oncology and Stem Cell Transplantation, Children’s Hospital, Helsinki University Hospital, Helsinki, Finland; ^14^Department of Pediatric Hematology and Oncology, Bambino Gesu Children’s Hospital, Sapienza, University of Rome, Rome, Italy; ^15^Department of Pediatric Oncology and Hematology, University of Bologna, Bologna, Italy; ^16^Department of Pediatric Hematology/ Oncology, Charles University and Univ Hospital Motol, Prague, Czech Republic; ^17^Department of Pediatric Oncology/Hematology, Skåne University Hospital, Lund, Sweden

Keywords: JMML, neurofibromatosis type 1, NF1

**Introduction:** Neurofibromatosis type 1 (NF-1) predisposes to juvenile myelomonocytic leukemia (JMML) via loss of *NF1* tumor suppressor function and consecutive deregulation of Ras signal transduction. Affected individuals carry one defective *NF1* allele in the germline; somatic inactivation of the second *NF1* allele in hematopoietic cells is associated with transformation to JMML. Here, we sought to clarify if genetic findings in leukemic cells of JMML/NF-1 patients were consistent with the two-hit concept. In addition, we asked if the group of JMML patients without clinical evidence of NF-1 and no mutation in *PTPN11*, *KRAS*, *NRAS*, or *CBL* (quintuple-negative, QN) contained unrecognized cases driven by *NF1*.

**Methods/ Results:** We investigated 156 children with JMML registered in studies EWOG-MDS 98 or 2006 and tested for mutations in *PTPN11*, *KRAS*, *NRAS*, and *CBL*. Twenty-five children (16%) were clinically diagnosed as NF-1 based on >=6 café-au-lait spots (CALS) or family history plus CALS. Sixteen children (10%) were grouped as JMML-QN. Granulocyte DNA from bone marrow or peripheral blood collected at time of diagnosis was used for next-generation sequencing of the entire *NF1* coding sequence (custom Ampliseq enrichment and Miseq, Illumina). Affymetrix Cytoscan HD arrays were applied to detect segmental deletions or copy number-neutral loss of heterozygosity. Among 25 JMML/NF-1 cases, 10 exhibited an *NF1* loss-of-function mutation at near-100% variant allelic frequency (VAF) in combination with uniparental disomy involving almost the entire 17q arm, suggesting single mitotic recombination as the leukemia-causing event. Eight cases carried two independent pathogenic *NF1* mutations at near-50% VAF each. In one of these cases, the two mutations were close enough to enable demonstration of in-trans configuration. However, germline and somatic events could not be distinguished in any of the 8 cases due to unavailability of non-hematopoietic or parental DNA. One case harbored three *NF1* mutations at 53%, 28%, and 13% VAF, suggesting two co-existing subclones. Four cases exhibited *NF1* microdeletions (3 typical, 1 atypical) in combination with a pathogenic *NF1* mutation at near-100% VAF. Non-hematopoietic tissue available in 3 of these 4 cases showed the microdeletion but failed to display the mutation. In two cases, monoallelic evidence of *NF1* deficiency (1 typical *NF1* microdeletion, 1 pathogenic mutation) but no second event was detected. In the JMML-QN group, 3 cases of compound-heterozygous pathogenic *NF1* mutations and 3 cases of microdeletions combined with hemizygous pathogenic *NF1* mutations were found. Non-hematopoetic tissue was available in one case and harbored the microdeletion but not the mutation. In the absence of clinical NF-1 features, the findings in these 6 children may be explained by 1) postzygotic *NF1* mosaicism, 2) onset of JMML before clinical manifestation of NF-1, or 3) double somatic *NF1* hits. Two JMML-QN cases carried single *NF1* mutations at VAF ~50% or less, providing inconclusive evidence of driver function. There was no genetic evidence of *NF1* involvement in the remaining 8 JMML-QN cases.

**Conclusions:** We conclude that the clinical diagnosis is reliable in children with JMML/NF-1, and propose that it can be made on the basis of CALS and JMML alone as most patients are too young to display the entire spectrum of NF-1 features. Genetic work-up of children without clinical features of NF-1 and no canonical driver mutation unmasks involvement of *NF1* in a significant number of cases. In suspected JMML-QN without identifiable *NF1* lesion, other myeloproliferative neoplasms should be considered.

## 34. CircRNAs Dysregulated in Juvenile Myelomonocytic Leukemia: CircMCTP1 Stands Out

### A. Dal Molin^1,*^, M. Hofmans^2,3*^, E. Gaffo^1^, H. Cavé^4^, V. de Haas^5,6^, C. Flotho^7^, J. Stary^8^, B. De Moerloose^2,9^, S. Bresolin^10,11^, S. Bortoluzzi^1,12^

#### ^1^Department of Molecular Medicine, University of Padova, Padova, Italy; ^2^Department of Pediatric Hematology-Oncology and Stem Cell Transplantation, Ghent University Hospital, Ghent, Belgium; ^3^Department of Diagnostic sciences, Ghent University Hospital, Ghent, Belgium; ^4^Department of Genetics, University Hospital of Robert Debré and Paris-Diderot University, Paris, France; ^5^Princess Máxima Center for Pediatric Oncology, Utrecht, The Netherlands; ^6^Dutch Childhood Oncology Group, The Hague, The Netherlands; ^7^Division of Pediatric Hematology and Oncology, Department of Pediatrics and Adolescent Medicine, University of Freiburg, Freiburg, Germany; ^8^Department of Pediatric Hematology/ Oncology, Charles University and University Hospital Motol, Prague, Czech Republic; ^9^Cancer Research Institute Ghent, Ghent, Belgium; ^10^Onco-hematology, stem cell transplant and gene therapy laboratory, IRP-Istituto di Ricerca Pediatrica, Padova, Italy; ^11^Department of Women’s and Children’s Health, University of Padova, Padova, Italy; ^12^Interdepartmental Research Center for Innovative Biotechnologies (CRIBI), University of Padova, Padova, Italy

* Equally contributing first authors

**Introduction:** Juvenile myelomonocytic leukemia (JMML) is a rare myelodysplastic/myeloproliferative neoplasm of early childhood and is characterized by clonal growth of RAS signaling addicted stem cells. JMML subtypes are defined by specific RAS pathway gene mutations and display distinct gene, microRNA (miRNA) and long non-coding RNA expression profiles. Recently, circular RNAs (circRNAs), single stranded RNA molecules with 3’- and 5’-ends backspliced in a non-collinear manner, were discovered, and shown to play key roles in many cancer types, including leukemia.

**Aim:** Our aim was to study the circRNA transcriptome in JMML and identify specific circRNA profiles associated with mutational subgroups and other disease characteristics.

**Methods:** Total RNA from diagnostic bone marrow (BM) or peripheral blood (PB) samples from 31 children with JMML and 8 healthy donors (HD) was obtained from different institutions throughout Europe. None of the patients received prior treatment. A discovery cohort of 19 JMML patients (8 *PTPN11*, 5 *KRAS*, 4 *NRAS*, and 2 *NF1* samples) and 3 HD was used for total paired-end RNA-sequencing profiling. CirComPara software, a bioinformatics pipeline which simultaneously uses 9 backsplice detection methods, was used to annotate and quantify circRNAs. Differential expression in pairwise comparisons between conditions or sample groups was assessed by DESeq2. Expression of 27 circRNAs was analyzed by qRT-PCR in an independent validation cohort of 12 JMML patients and 6 HD. CircRNA-miRNA-gene networks were reconstructed using custom circRNA function prediction and gene expression data.

**Results:** Circular RNAs expression profiles separated JMML samples from HD according to unsupervised principal component analysis, pointing toward a general dysregulation of circRNA expression in patients. Considerable heterogeneity was observed among JMML patients, and both PCA and clustering based on circRNA expression showed that *KRAS*, *NRAS*, and *PTPN11* mutated patients clustered together, while the two *NF1* samples clearly separated from both HD and the other molecular groups. We identified 119 circRNAs dysregulated in JMML, of which 90 up- and 29 down-regulated. Of those, 37 circRNAs had different expression levels in the entire JMML cohort compared to HD, whereas the others were only dysregulated in one or more molecular subgroups. Our data indicated also circRNA expression differences among molecular subgroups of JMML. Analysis in the validation cohort of 27 circRNAs with dysregulated expression in JMML confirmed the downregulation of circOXNAD1 and circATM, and a marked up-regulation of circLYN, circAFF2, and circMCTP1. Interestingly, we identified upregulated circMCTP1 to be linked to expression level of known tumor suppressor miRNAs in JMML.

**Conclusions:** In conclusion, this study provides insight into the circRNA transcriptome of JMML. Dysregulation of known and many unknown circRNAs, such as circMCTP1, suggests a role for circRNAs in JMML pathogenesis, and results of functional predictions indicated the genes and biological pathways possibly involved. This study paves the way for functional research on the impact of circRNA dysregulation in JMML biology and their diagnostic and therapeutic applications.

## 35. Immunophenotypic changes in JMML patients treated with hypomethylating agents. A correlation with clinical response assessment

### A. Frisanco Oliveira^1^, A. Tansini^1,2^, T.R. Toledo^1^, R. Balceiro^1,2^, N. Villela^1,2^, L. Fernando Lopes^1,2^, I. Lorand Metze^2,3^

#### ^1^Barretos Children Cancer Hospital, Barretos, Brazil; ^2^Brazilian Cooperative Group of Pediatric Myelodysplastic Syndrome, Barretos, Brazil; ^3^Hemocentro Hospital das Clinicas Unicamp, Campinas, Brazil

##### **Correspondence:** A. Frisanco Oliveira

**Introduction:** 5-azacitidine has been considered to be safe and efficient medication for use as a bridge to hemotopoietic stem cell transplantation in JMML patients. Patients under clinical treatment can be stratified as complete or partial response, stable disease or disease progression. Recently, the features found in JMML immunophenotyping (FCI) at diagnosis have been described and so, they could be useful for examining these characteristics for determining response of the patients to treatment.

**Aim:** to follow the immunophenotypic pattern of JMML patients during treatment with 5-azacitidine, with an already validated IFC protocol and also look for cytometric features that correlate with the type of clinical response.

**Methods:** An 8-color antibody panel described for pediatric population with JMML at diagnosis was used. Patients treated with cycles of 5-azacitidine 75mg/m^2^ subcutaneous for consecutive 7 days, each 28 days were evaluated at diagnosis and after 3 and 6 cycles of medication. The findings were compared with the type of clinical response.

**Results:** 32 patients entered the study. Among them, only 28 patients could be analyzed by FCI after 3 cycles and 25 patients after 6 cycles. After treatment with 5-azacitidine, patients showed a reduction in CD34^+^ cells, with median CD34/CD117^+^ cells going from 3.35% to 2.8% after 3 cycles of medication. Values dropped to <2% in 12 and 22 patients after 3 and 6 cycles respectively, and this was observed only in patients responding to treatment. B cell progenitors (H1), that were decreased at diagnosis, had a further reduction after treatment. The monocytic population also decreased, from 11.91% to 7.5% and 6.4% after 3 and 6 cycles, respectively. Those presenting with complete response had an apparent increase in classical monocytes and a decrease in intermediate monocytes. Finally, the population of T lymphocytes, that were reduced at diagnosis, showed an important increase, especially in patients with a clinical response (p <0.001); an increase of NK lymphocytes was also observed mainly after 3 cycles from 1.01% to 1.85%. Immunophenotypic changes including aberrant expression of CD7 in myeloid progenitors remained after 3 and 6 cycles of 5-aza. Aberrant CD7 expression in myeloid progenitors was associated with a worse response to treatment, as well as NF1.

**Conclusions:** FCI was a feasible tool in JMML patients after treatment with 5-azacitidine. Clinical response was associated with decrease of CD34^+^ myeloid progenitor and total monocytes and a rise in T and NK lymphocytes. But abnormal co-expressions remained, even in patients with complete clinical response and this speaks in favor of the persistence of the leukemic clone during the whole clinical treatment. The largest effect was seen after 3 cycles. CD34/117^+^ cell and T lymphocytes are variables that could be incorporated into the assessment of response to clinical treatment of JMML patients. A more detailed study of the several subsets of T cells should be included. The same MFC protocol could be used pre and post transplantation identifying patients with beneficial use of 5-aza after transplant, as it has recently been shown according to methylation status. Prospective studies are necessary to confirm the data with a larger number of patients, and including assessment of the pre and post transplantation status.

## P1. Myelodysplastic syndrome in two siblings with del 16q22 and family history of neutropenia

### R. Balceiro^1,2^, A. Frisanco Oliveira^1,2^, N. Costa Villela^1,2^, H. de Campos Reis Galvão^2^, R. de Souza Ferreira^1^, V. de Oliveira Viana^2,3^, L. Fernando Lopes^1,2^

#### ^1^Barretos Children’ Cancer Hospital, Barretos, Brasil; ^2^Brazilian Cooperative Study Group for Pediatric Myelodysplastic Syndrome (GCB-SMD-Ped) , Barretos, Brazil; ^3^Albert Sabin Children’s Hospital, Fortaleza, Brazil

##### **Correspondence:** R. Balceiro

**Introduction:** Deletion of 16q22 is rare in myeloid neoplasms and was considered a variant form of inv(16)(q22). However, recent data showed that AML with del(16)(q22) has different clinical findings and worse prognosis. On the other hand, del(16)(q22) has also been described in familial cases of benign neutropenia.

**Methods/Cases:** Case 1: female, 10 years, with neutropenia since age of 6. Biological mother with history of drug abuse (had 6 children). Half-sister by mother (16 years-old) with neutropenia. On physical examination: mild facial dysmorphism. Hemoglobin 11.8g/dL, hematocrit 35.4%, leukocytes 2.2x10^9^/L (neutrophyls 0.61, lymphocytes 1.43, monocytes 0.11) Platelets 337x10^9^/L. Normal serologies. Normal B12 and folic acid levels. Normal fetal hemoglobin. Bone marrow aspiration (BMA) with dyseritropoiesis, dysplastic megakaryocytes and 3% of blasts. Immunophenotyping with 1,5% of myeloid precursors without aberrant expression. Bone marrow biopsy (BMB) hypocelular (40%) with dysplastic features. Next-generation sequencing myeloid panel negative. Karyotype: 46, XX, del(16)(q22) [8]/47, idem,+mar [2]/46, XX [10]. FISH for MYH11/CBFB with deletion of CBFB in 43% of cells. Diagnosis: MDS-Refractory Cytopenia of Childhood. After 6 months stable, new BMA were performed and showed dysplastic features and 7% of myeloid blasts. Due to evolution to MDS with excess blasts, she underwent hematopoietic stem cell transplantation from an unrelated matched donor. Received myeloablative conditioning regimen composed of Busulfan, Cyclophosphamide and Melphalan. Neutrophilic engraftment on D+27. On D+100, she evolved with chronic graft-versus-host disease of the gastrointestinal tract. Currently, she is on D+229 post-transplant, in medullary remission and with complete chimerism.

Case 2: female, age 16, with neutropenia since 15 years. Asymptomatic. Biological mother with history of drug abuse (had 6 children). Half-sister by mother (10 years-old) with MDS (case 1). Physical examination: mild facial dysmorphism. Hemoglobin 11.9g/dL, hematocrit 37%, leukocytes 2.2x10^9^/L (neutrophyls 0.62, lymphocytes 1.43, monocytes 0.11), platelets 337x10^9^/L. Normal serologies, B12, folic acid levels, fetal hemoglobin. BMA hypocellular with dyseritropoiesis and dysgranulopoiesis with 2,6% of blasts. Immunophenotyping with 1,05% of myeloid precursors without aberrant expression. BMB hypocelular (30%) with dysplastic megakaryocytes. Karyotype: 46, XX, del(16)(q22)[3]/46, XX, fra(16)(q22)[3]/46, XX[14]. Diagnosis: MDS-Refractory Cytopenia of Childhood. After almost 2 years of follow-up with watch and wait approach, she remains asymptomatic, with no signs of disease progression.

Mother and 7 year old sibiling also have neutropenia, but she refused to be tested for del16)(q22).

**Results:** Literature reports family cases of del(16)(q22) and benign neutropenia with affected individuals remaining asymptomatic during years/decades. However, these patients don’t show dysplastic features on BMA. In contrast, this cytogenitic finding also correlates with AML/MDS, and such cases have more severe course than AML with inv(16)(q22), showing deletion of CBFB gene just like case 1. The disease progression in patient 1 suggests an aggressive disease related to del(16)(q22) in this family.

**Conclusions:** Due to the rarity of myeloid neoplasms with del(16)(q22), little is known about its characteristics. Moreover, this deletion is also related to benign neutropenia, with no need for intervention. Therefore, it is necessary a close follow up of these patients in order to define the best treatment.

Consent to publish has been obtained.

## P2. Emberger syndrome and GATA2 deficiency in 2 adolescents with advanced myelodysplastic syndrome - case report

### R. Balceiro^1,2^, A. Frisanco Oliveira^1,2^, N. Costa Villela^1,2^, H. de Campos Reis Galvão^2^, R. de Souza Ferreira^1^, L. Fernando Lopes^1,2^

#### ^1^Barretos Children’ Cancer Hospital, Barretos, Brazil; ^2^Brazilian Cooperative Study Group for Pediatric Myelodysplastic Syndrome (GCB-SMD-Ped), Barretos, Brazil

##### **Correspondence:** R. Balceiro

**Introduction:** *GATA2* gene encodes a key transcription factor for normal hematopoiesis. It is highly expressed in immature hematopoietic cells, being crucial for proliferation and maintenance of the stem-cell pool. Several manifestations make up the spectrum of *GATA2* deficiency (immunodeficiency, MDS, lymphedema, mycobacterial infections).

**Methods/Case report:** Patient 1, age 17, male, history of 6 months with recurrent cellulitis in legs and local edema associated with pancytopenia. Bilateral hearing loss since 9 years. On physical examination, lymphedema in left leg and warts on his hands and arms. Hemoglobin 9.7 g/dL, hematocrit 31.4%, leukocytes 9.3x10^9^/L (neutrophils 3.72 lymphocytes 4.371, monocytes 0.372), platelets 764 x10^9^/L. Myelogram: normocellular with severe dyspoiesis and 8% blasts. Immunophenotyping: 7.9% CD34/CD117 cells, with abnormal expression of CD7 and CD36. Absence of B cell precursors. 0.16% B lymphocytes, 1.67% NK and 7.06% T lymphocytes, 0.13% monocytes. Bone marrow biopsy: 90% cellularity, with severe megakaryocytic dysplasia. Karyotype: 45,XY,-7[20]. Next-generation Sequencing (NGS): mutations in *ASXL1, SETBP1, GATA2.* Final diagnosis: myelodysplastic syndrome with excess blasts and *GATA2* deficiency with Emberger syndrome. He underwent hematopoietic stem cell transplantation (HSCT) from a haploidentical donor (father), with a myeloablative conditioning regimen consisting of busulfan, fludarabine and melphalan. Neutrophilic engraftment on D+18. The patient developed severe chronic graft-versus-host disease with involvement of the skin, mouth, eyes and gastrointestinal tract, refractory to therapy. Death on D+389 due to infectious complications.

Patient 2, age 15, male, with anemia since 10 years. In 2018 he developed pancytopenia. On physical examination, edema in legs and warts on his hands. Hemoglobin 9.3 g/dL, hematocrit 30.4%, leukocytes 0,8 x10^9^/L (neutrophils 0.352, lymphocytes 0.24, monocytes 0.104), platelets 4 x10^9^/L. Myelogram: hypocellular with trilineage dysplasia and 13.8% of blasts. Immunophenotyping: 14.4% CD34/CD117+ cells without abnormal expressions. Absence of B precursors. 1.43% of B lymphocytes, 2.17% of NK and 6.27% of T lymphocytes, 1.66% monocytes. Bone marrow biopsy: 100% cellularity with severe megakaryocytic dysplasia. Karyotype: 45,XY,-7[17]/45,del(5)(q22q35)[3]. NGS: mutations in *ASXL1, SETBP1, GATA2* and VUS (variant of unknown significance) in *IDH1*. Final diagnosis: myelodysplastic syndrome with excess blasts and *GATA2* deficiency with Emberger syndrome. He started azacitidine while waiting for bone marrow transplantation, but died due to severe infection during.

**Discussion:** Both patients had *GATA2* deficiency with Emberger syndrome (MDS and lymphedema) and monosomy of 7, as well as mutations in *ASXL1* and *SETBP1*. The immunophenotyping of both is characteristic, with a reduction in precursors and B lymphocytes, in addition to monocytopenia. Somatic mutations in *ASXL1* and *SETBP1* are recurrent in patients with MDS and *GATA2* deficiency. It is believed that they can contribute to clonal evolution in the context of leukemic transformation. Both cases were diagnosed with advanced MDS. Monosomy 7 is the main cytogenetic alteration found in these patients, especially adolescents. Bone marrow transplantation is the only curative option for patients with GATA2 deficiency.

**Conclusions:** *GATA2* deficiency is a rare, heterogeneous clinical condition. Early detection is important due to the risk of progression to AML, and bone marrow transplantation is the only curative option available.

Consent to publish has been obtained.

## P3. Introduction of HPO annotations for enhanced identification of genotype-phenotype correlations and phenotypic disease spectra in IBMFS

### J.Berner^1,2,3^, D. Mayr^1,2,3^, M. Haimel^1,3,4^, W. Novak^2^, J. Pazmandi^1,3,4^, V. Hertlein^1,3,4^, M. Dworzak^1,2^, L. Kager^2^, K. Boztug^1,2,3,4,5^

#### ^1^St. Anna Children’s Cancer Research Institute (CCRI), Vienna, Austria; ^2^St. Anna Children’s Hospital, Department of Pediatrics and Adolescent Medicine, Medical University of Vienna, Vienna, Austria; ^3^Ludwig Boltzmann Institute for Rare and Undiagnosed Diseases, Vienna, Austria; ^4^CeMM Research Center for Molecular Medicine of the Austrian Academy of Sciences, Vienna, Austria; ^5^Department of Pediatrics and Adolescent Medicine, Medical University of Vienna, Vienna, Austria

**Introduction:** Precise and standardized phenotypic descriptions are of utmost importance to establish genotype-phenotype relations in rare diseases. Further, detailed and accurate descriptions are necessary to help geneticists and clinicians interpret functional relevance of genetic variants in rare hematologic disorders. The Human Phenotype Ontology (HPO) offers a unique catalogue of standardized phenotypic features in a hierarchical structure that is gaining interest in rare disease research. Yet, until today the applications in pediatric hematology especially with the focus on inherited Bone Marrow Failure syndromes (iBMFs) are scarce. IBMFS comprise a heterogeneous group of genetic disorders that share a malfunctioning bone marrow with a large spectrum of cytopenias, physical malformations and an increased risk of myelodysplastic syndromes as well as other malignancies. Within this group of diseases multiple gene defects have been identified, but a significant proportion of cases still remains genetically unsolved. Further, even patients with similar genetic defects show different clinical courses as well as treatment responses. Essentially, a clear genotype-phenotype relation is missing, and the phenotypic complexity amplifies diagnostic challenges.

**Aims:** This study aims at using HPO terminology, protein-protein interaction (PPI) networks and deep clinical phenotyping to investigate genotype-phenotype relations in patients with iBMFs. Further, this study aims to improve the diagnostic efforts and variant calling, through phenotypic comparison of genetically unsolved with solved cases.

**Methods:** We use network analysis for PPIs to investigate the proximity of known iBMF genes. Additionally, we systematically reviewed HPO diseases annotation for iBMFS and compared them to other rare diseases. Further, we performed deep clinical phenotyping to computationally align and compare different patient subgroups.

**Results:** By comparing proximity of proteins encoded by known causative iBMF genes, we have found that these genes cluster significantly closer than expected by chance within our PPI network. Further, we have shown that iBMFs are sufficiently annotated with HPO terms and thus are suitable for bioinformatic application. Additionally, we have shown that the annotation of phenotypic features significantly improves disease ranking in rare diseases. We thus have annotated phenotypic features of a preliminary set of local patients with HPO terms and performed unsupervised clustering of patient subgroups. By comparing clinical groups with PPI clustering and pure phenotyping, we observed partial overlap between subgroups, yet due to a limited dataset, we were not able to cluster the unsolved cases.

**Conclusions:** Our computational approach of combined network analysis and deep clinical phenotyping presents a feasible, objective and structured method to investigate genotype phenotype relations in iBMFS.

## P4. Molecular profile and survival of patients with Juvenile Myelomonocytic Leukemia subjected to hematopoietic stem cell transplantation in a single Brazilian center

### R. De Souza Ferreira^1^, P. Shimoda Ikeuti^2,3^, N. Costa Villela^1,3^, D. Onofre Vidal^3^, S. Frahia Bento da Silva^1,3^, J. Costa Gaspar^1,3^, L. Fernando Alves Lima Mantovani^1^, A. Mandelli Venancio^1^, G. Maurício Navarro Barros^1^, L. Fernando Lopes^1^

#### ^1^Barretos Cancer Hospital, Barretos, Brazil; ^2^Instituto de Oncologia Pediátrica IOP/GRAACC/Unifesp, São Paulo, Brazil; ^3^Brazilian Pediatric Myelodysplastic Syndrome Study Group, Barretos, Brazil

##### **Correspondence:** R. De Souza Ferreira

**Introduction:** Juvenile Myelomonocytic Leukemia (JMML) is an aggressive hematopoietic disorder of infancy/early childhood. In about 90% of cases, patients present somatic or germline mutations in one of five genes (*PTPN11*, *KRAS*, *NRAS*, NF1 and *CBL*) which define genetically and clinically distinct subtypes. Although few cases show spontaneous remission, allogeneic hematopoietic stem cell transplantation (HSCT) remains the only curative treatment option for the majority of patients.

**Aims**: To describe the molecular profile of JMML patients subjected to allogeneic HSCT in the pediatric transplant unit of the Barretos Cancer Hospital. To evaluate the survival rates after HSCT of the total cohort and according to the different molecular mutations.

**Methods:** All the 27 patients diagnosed with JMML who underwent their first HSCT at our center, between January 2014 and July 2019, were selected for this retrospective study. Along with descriptive analysis, a measurement on the global survival and event-free survival of the total cohort and of each molecular marker group was done. The survival probability was estimated by a Kaplan-Meier curve and the comparisons were made by the Logrank test. The chosen significance level was 0.05 and the data was analyzed by the SPSS software, version 2.1.

**Results:** Nine patients (33%) had *PTPN11* mutation, four (15%) had *KRAS*, four (15%) had *NRAS*, one (4%) had both *PTPN11* and *NRAS* mutations, six (22%) had been clinically diagnosed with neurofibromatosis type 1 (NF1) and 3 (11%) had no molecular markers. No germline mutations have been documented. Considering all analyzed patients, the 3-year overall survival (OS) and event-free survival (EFS) was 65% and 59.3%, respectively. Relapse was the major cause of death. According to the genetic subgroup, the estimate 3-year OS was: 61% for PTPN11-mutated JMML, 100% for NRAS, 50% for KRAS, 50% for patients diagnosed with NF1 and 100% for patients with no molecular markers (P=0,153). The patient with simultaneous mutations of *PTPN11* and *NRAS* died from disease relapse.

**Conclusions:** PTPN11 mutation was the most common in our series, similar to that reported in the literature, however the proportion of children with NF1 was relatively high among our patients. We found no difference in survival between the different genetic subgroups, most likely due to the small sample size.

## P5. Identification of cooperating factors promoting leukemogenesis in individuals with GATA2 syndrome

### C. Frank^1^, J. Fernandez-Orth^1^, J. Miriam Weiss^1^, G. Andrieux^2^, Venugopal R. Mittapalli^1^, S. Bohler^1^, C. Molnar^1^, C. Niemeyer^1^ and M. Erlacher^1^

#### ^1^University Medical Center Freiburg, Center for Pediatrics Department of Pediatric Hematology and Oncology, Freiburg, Germany; ^2^Institut für Medizinische Bioinformatik und Systemmedizin Medizinische Fakultät, Albert-Ludwigs-Universität Freiburg

**Introduction:** It controls gene transcription from multiple target genes by binding to the consensus DNA sequence (T/A(GATA)A/G) of many enhancers, suppressors and promotors. More than 400 different germline mutations have been already identified in GATA2, including missense, frameshift and splice-site-mutations within the zinc finger 2 (ZF2) domain as well as non-coding substitutions in the EBOX-GATA-ETS. This variability leads to a very complex and heterogeneous syndrome, characterized by variable cytopenias as well as immunodeficiency, lymphedema or deafness among other clinical problems. Even more, the risk of affected individuals to develop either MDS or AML is exceedingly high, however, the mechanisms underlying malignant transformation are only insufficiently studied.

**Methods/Results:** Here, we hypothesize that predisposition mutations can lead to cellular deficiencies that are compensated by somatic events. Such somatic events, however, might be associated with oncogenic transformation. For this purpose, it is important to identify the genes and pathways affected by GATA2 haploinsufficiency and to analyze how their activation or inhibition, respectively, affects HSPC function and leukemogenic potential. Using Gata2^fl/+^ LSK cells (Lin^-^, Sca-1^+^, c-Kit^+^) treated with recombinant CRE (i.e. TAT-CRE) to delete one *Gata2* allele and forced to proliferate *in vitro*, we performed RNA-seq to analyze the transcriptome and compare them to WT cells treated accordingly. Among other genes, many MYC targets were found to be downregulated in GATA2^+/-^ cells compared to WT cells. Since MYC is a crucial transcription factor that plays a role in the generation and maintenance of the hematopoietic system, we are studying whether lentiviral MYC overexpression *in vitro* in Gata2^+/-^ cells is sufficient to compensate the defective stress response in Gata2^+/-^ HSPCs. This will be analyzed in apoptosis, proliferation and colony forming assays.

**Conclusions:** We hypothesize that MYC overexpression can positively affect survival, proliferation and differentiation of HSPCs.

## P6. Pediatric myelodysplastic syndrome with chromosome 7 alterations: a Portuguese single centre experience

### J. Gaião Santos^1^, I. Luz^2^, H. Vitória^3^, M. Jerónimo^2^, M. Brito^2^, M. Coucelo^1^, I. Ferreira^4^, A. Campos^5^, J. Azevedo^1^, A. Crisóstomo^1^

#### ^1^Serviço de Hematologia Clínica, Centro Hospitalar Universitário de Coimbra, Coimbra, Portugal; ^2^Serviço de Oncologia Pediátrica, Centro Hospitalar Universitário de Coimbra, Coimbra, Portugal; ^3^Serviço de Hematologia, Centro Hospitalar Tondela-Viseu, Viseu, Portugal; ^4^Unidade de Transplante de Medula, Instituto Português de Oncologia de Lisboa, Lisboa, Portugal; ^5^Serviço de Transplantação de Medula Óssea, Instituto Português de Oncologia do Porto, Porto, Portugal

##### **Correspondence:** I. Luz

**Introduction:** Myelodysplastic syndromes (MDS) are rare in children (<5% of hematological malignancies). Monosomy of chromosome 7 (─7) or 7q deletion (del(7q)) are found in >20% and associated with a very poor outcome. We present 3 children with MDS and chromosome 7 aberrations.

**Methods/Case series:** Case 1: 9yo boy was referred due to mild thrombocytopenia. During follow-up thrombocytopenia worsened and he developed mild neutropenia. Bone marrow (BM) evaluation was performed: hypocellular BM (<10% cellularity) with no dysplastic features nor excess blasts; karyotype: 46,XY,inv(9)(p12q13)[20] described as a non-pathological variant; FISH analysis for del(5q), -7, del(7q), +8, del(20q), -Y was normal. Given worsening blood counts (Hb 7.9g/dL, retic 17x10^9^/L, neut 0.52x10^9^/L, plat 11x10^9^/L) and exclusion of other entities he was diagnosed with severe aplastic anemia. No related HLA matched donor was found so he was treated with horse ATG + cyclosporine achieving partial remission and transfusion independence at 6 months.

BM assessment after 1 year of treatment revealed: heterogeneous cellularity with erythroid dysplasia, <10% myeloid dysplasia, and a new karyotype aberration involving cr 7: 46,XY,der(7;9)(q10;p10),+9,inv9(p12q13)c[2]/46,XY,inv(9)(p12q13)[18]. He was diagnosed with refractory cytopenia of childhood (RCC) and referred for unrelated donor hematopoietic stem cell transplantation (HSTC) achieving complete remission (CR) which he maintains.

Case 2: 5yo girl referred due to pancytopenia (Hb 10.3g/dL, MCV 99fL, retic 83x10^9^/L, neut 1.1x10^9^/L, plat 143x10^9^/L). She had mild psychomotor development delay, microcephaly and hirsutism, but known BM failure syndromes (BMFs) such as Fanconi Anemia, SBDS or DBA were excluded. BM evaluation: normocellular, trilineage dysplasia and no excess blasts. BM karyotype: 45,XX,-7[9]/46,XX[4]. She was diagnosed with MDS with ─7 and underwent unrelated donor HSTC, achieving CR. She had an unrelated death 3 years later while in CR.

Case 3: 4yo boy presenting with fever and pancytopenia (Hb 8.7g/dL, MCV 92fL, retic 28x10^9^/L, neut 1.0x10^9^/L, plat 113x10^9^/L). After excluding other causes, BM evaluation was performed: normocellular BM with dysplasia and 7% blasts. BM karyotype showed 45,XY,-7[20], confirmed by FISH. A customized NGS panel for BM failure and somatic myeloid variants showed 3 low frequency (VAF <50%) somatic mutations in *RUNX1*, *PTPN11* and *NF1*. No germline variants or known predisposing conditions were found. He was diagnosed with MDS with excess blasts and ─7. Matched sibling donor HSCT was performed 3 months later, achieving CR. The patient had an early relapse (3 months) with progression to acute myeloid leukemia (AML) in spite of DLI, accompanied by refractory hepatic GVHD. He was scheduled for salvage therapy with azacytidine, but died of infectious complications before starting treatment.

**Conclusions:** Pediatric MDS is associated with inherited BMFs or genetic predisposition syndromes in >30% of cases. We did not identify any known germline predisposing features in our small series. Given the very high risk of AML progression in monosomy 7 and 7q deletion HSCT remains the only curative therapy. Evolving high throughput technologies can help us better understand the nature of these complex and heterogeneous disorders and, together with novel therapeutic agents, allow us to improve therapeutic strategies.

Consent to publish has been obtained.

## P7. Morphological Distinction Between Aplastic Anemia and Hypocellular Refractory Cytopenia of Childhood

### C. Kelaidi^1^, G. Mitropoulou^2^, K.Tsitsikas^1^, K. Antoniadi^1^, A. Bountali^1^, L. Petrikkos^1^, K. Stefanaki^2^, S. Polychronopoulou^1^

#### ^1^Department of Pediatric Hematology and Oncology, “Aghia Sophia” Children’s Hospital, Athens, Greece; ^2^Department of Pathology, “Aghia Sophia” Children’s Hospital, Athens, Greece

##### **Correspondence:** C. Kelaidi

**Introduction:** The distinction between aplastic anemia (AA) and hypoplastic refractory cytopenia of childhood (h-RCC) in the absence of clonality markers is based on morphological criteria on bone marrow (BM) aspiration smears and biopsies.

**Aim:** To assess hypocellularity and dysplasia in AA in distinction to h-RCC on BM smears and biopsies according to EWOG criteria.

**Methods:** BM smears of children and young adults diagnosed in a single center from 2009 to 2019 with hypocellular BM failure syndrome were centrally reviewed by two examinators and compared with their concomitant BM biopsies. A score of hypocellularity was used on smears and biopsies, with values of 0, 1, 2, 3 and 4 indicating empty BM, severe, moderate, mild reduction of cellularity and normal cellularity, respectively. Definitive diagnosis of AA vs. h-RCC was based on all available data including morphology, cytogenetics, mutations, syndromic features, clinical presentation and course of the disease. Fanconi anemia was excluded in all cases.

**Results:** Overall, we examined smears from 25 patients: 47 smears from 10 AA and 15 h-RCC patients at diagnosis, and 8 smears following immunosuppressive therapy (IST) from 5 AA patients. Median number of smears per patient was 2 (range 1-6). Male/female ratio was 21/4 (84%) and median age 6.8 y (range 1-21). AA vs. h-RCC patients had median values of neutrophils 0.44 vs. 1.2 G/L (P=0.04), Hb 8.2 vs. 10.7 g/dL (P=0.14), platelets 19 vs. 67 G/L (P=0.08), respectively. h-RCC was associated with genetic syndromes in Cornelia de Lange N=1, SAMD9/SAMD9L N=2, dyskeratosis congenita N=1. Karyotype was -7 N=1, +21 N=1, normal N=23. Cellularity was 0/1/2/3/4 in 35%, 18%, 18%, 11% and 18% of BM smears, respectively. Overall, 80% of AA vs. 40% of h-RCC patients had ≥1 smear of null cellularity, respectively (P=0.11). Erythroid (E), granulocytic (G) and megakaryocytic (M) lineage was absent in 33%, 46% and 87% of AA patients, and in 9%, 9% and 16% of h-RCC patients, respectively. Increased mast cells were present in 47% of all patients, respectively, without difference between AA and h-RCC. Asynchronous nuclear and cytoplasmic maturation and double/triple nuclei were the more frequent signs of dyserythropoiesis. Dysgranulopoiesis with hypogranulation was observed only in h-RCC patients. Micromegakaryocytes and megakaryoblasts were rare abnormalities as compared with less specific changes of dysmegakaryopoiesis, like nuclear immaturity and abnormal maturation. Histology was assessed on 39 concomitant biopsies from 9 AA patients and 12 h-RCC patients. Severely reduced cellularity was more frequent in AA than in h-RCC (100% vs. 38% of patients, respectively, P=0.01). At least one occurrence of paratrabecular erythroblastic islands and micromegakaryocytes was observed in 33% vs. 100% (P=0.06) and 0 vs. 58% (P=0.04) of AA and h-RCC patients, respectively. Diagnostic discordance was noted between consecutive smears or between smear and concomitant biopsy in 2 and 1 patient, respectively, all with a final diagnosis of h-RCC based on histological evidence of erythroblastic islands. BM morphological and clinical/hematological response to IST, were concordant in AA patients with no response N=1, relapse N=1, and reponse N=3. Cellularity remained mildly reduced in responders beyond two years after IST.

**Conclusions:** h-RCC is characterised morphologically by less profound hypocellularity and more important dysplasia as compared to AA with, however, some important, in terms of diagnostic accuracy, overlap between these two distinctive BM failure syndromes.

## P8. The cytogenetic profile of 14 pediatric patients with MDS by conventional and molecular cytogenetic analysis

### K.N. Manola^1^, P. Diamantopoulou^1^, C. Kelaidi^2^, L. Petrikkos^2^, K. Stefanaki^3^, E.D. Ioannidou^4^, N. Tourkantoni^5^, K. Tsitsikas^2^, M. Kourti^6^, K. Antoniadi^2^, H. Tsipou^5^, E. Dana^7^, I. Pelagiadis^8^, E. Mantadakis^9^, M. Economou^10^, A. Makis^11^, E. Papakonstantinou^6^, H. Kosmidis^7^, E. Stiakaki^8^, G. Paterakis^12^, A. Kattamis^5^, I. Peristeri^4^, M.I. Margariti^1^, D. Pantelia^1^, C. Sambani^1^, S. Polychronopoulou^2^

#### ^1^Laboratory of Health Physics, Radiobiology and Cytogenetics, National Center for Scientific Research Demokritos, Athens, Greece; ^2^Department of Paediatric Haematology-Oncology, Aghia Sophia Children’s Hospital, Athens, Greece; ^3^Department of Pathology, Aghia Sophia Children’s Hospital, Athens, Greece; ^4^Bone Marrow Transplantation Unit,«Aghia Sophia Children’s Hospital, Athens, Greece; ^5^Division of Paediatric Hematology-Oncology, 1st Dpt of Pediatrics, National & Kapodistrian University Of Athens, Aghia Sophia Children’s Hospital, Athens, Greece; ^6^Departments of Paediatric Oncology, Hippokration General Hospital, Thessaloniki, Greece; ^7^Pediatric & Adolescent Oncology Clinic, “Mitera” Hospital, Athens, Greece; ^8^Department of Paediatric Hematology-Oncology, University Hospital of Heraklion, Heraklion, Crete, Greece; ^9^Department of Pediatrics, Democritus University of Thrace, University General Hospital of Alexandroupolis, Alexandroupolis, Greece; ^10^Division of Haematology, 1st Dpt of Paediatrics, Aristotle University of Thessaloniki, Hippokration General Hospital, Thessaloniki, Greece; ^11^Department of Paediatrics, University of Ioannina, University Hospital, Ioannina, Greece; ^12^Department of Immunology, Peripheral General Hospital Athens Giorgos Gennimatas, Athens, Greece

**Introduction:** Pediatric myelodysplastic syndromes (MDS) are a rare group of clonal hematopoietic stem cell disorders accounting for less than 5% of hematopoietic neoplasia in childhood with an incidence of 2–4 cases/million.

**Aim:** The aim of the study was to identify chromosomal abnormalities in pediatric patients with MDS, including submicroscopic aberrations such as gene alterations associated with the disease and possibly malignant transformation by conventional (karyotype) and molecular cytogenetic (FISH) analysis.

**Methods:** This study includes 14 children with MDS from 2015 until 2019. Karyotypic analysis was performed on 24h, 48h and 72h unstimulated bone marrow cultures using GTG-banding. Karyotypic analysis was carried out on 25 at least metaphases and karyotypes were described according to the international system for human cytogenomic nomenclature (ISCN) 2016. Molecular cytogenetic analysis was performed on cultured bone marrow cells by fluorescence in situ hybridization (FISH) using commercial probes for the centromere (CEP) of chromosomes 7 and 8 and the chromosomal regions 7q22, 7q31, 8q24(MYC), 11q22.3(ATM), 11q23(MLL), and 17p13(TP53). For each sample at least 200 interphase cells (iFISH) were analyzed. Normal cut-off was estimated at 3%.

**Results:** The sex ratio was 1,33 (8 males/6 females) and the mean age was 9,23 years (range 0,4y -16,8y). Nine patients had MDS-RCC and 5 MDS-EB. The karyotypic analysis was successful in all patients showing a normal karyotype in 8 patients (57,1%) and an abnormal karyotype in 6 patients (42,9%).

Monosomy 7 was observed in 2/14 patients (in 21/30 of metaphases in one and in 30/30 metaphases of the other), inv(17)(p11.2q21) in 1/14 patients in 3/16 metaphases, trisomy 8 in 2/14 patients (in 1/30 metaphases in one patient confirmed by FISH and in 1/40 in the other patient confirmed by one more metaphase by FISH) and a complex karyotype 46,XΥ,t(4;17)(p16;q23)[17]/46,Υ,add(X)(p22),del(2)(q33),add(4)(q21),der(7)del(7)(p15)t(4;7)(q21;q32)[3]/46,XY,add(5)(p11),-10,+mar[2]/46,XY[11] in 1/14 patients after BMT in the transformation of MDS into s-AML. FISH revealed an abnormal pattern for CEP7, 7q22, 7q31 in 2/14 patients confirming monosomy 7 in karyotype, one signal (copy) for p53 in 2/14 patients in low frequency (6,5% and 3,5% of the analyzed cells), both with hypocellular RCC and transfusion-dependency, that underwent BMT, 3 signals (copies) for CEP8 in 2/14 patients (in 8% and 2,7% of the analyzed cells) confirming trisomy 8 found in their karyotypes and one copy or rearrangement of MLL gene in the patient that was transformed to s-AML in 4,4% of the analyzed cells. A normal pattern of hybridization was observed for the above probes in the rest cases and for MYC and ATM genes in all the examined cases.

**Conclusions:** Chromosome aberrations were detected in 42,9% of children with MDS including monosomy 7, trisomy 8, inv(17) and a complex karyotype. Clonal chromosome abnormalities can be detected at a low frequency in the diagnostic karyotypes of pediatric MDS patients. The low frequency of submicroscopic aberrations such as p53 deletions observed in two cases of hypocellular RCC in this study must be confirmed in larger studies by molecular analysis as they may play a potential role in MDS development.

## P9. Allogeneic Hematopoietic Stem Cell Transplantation (HSCT) in Childhood Myelodysplastic Syndrome (MDS) – The Greek Experience

### C. Oikonomopoulou, A. Paisiou, A. Komitopoulou, E.-D. Ioannidou, A. Kaisari, M. Kastamoulas, G. Stavroulaki, G. Vessalas, E. Goussetis, I.Peristeri

#### Stem Cell Transplant Unit, Aghia Sophia Children’s Hospital, Greece

##### **Correspondence:** C. Oikonomopoulou

**Introduction:** Myelodysplastic syndromes comprise a heterogeneous group of clonal disorders and are rare in children. 30%-45% of pediatric MDS is associated with an underlying genetic predisposition syndrome and a subset of patients presents with secondary MDS following chemotherapy for an unrelated malignancy. HSTC is commonly used in children with MDS.

**Aim**: Our aim was to retrospectively examine the outcome of children with MDS transplanted in our unit.

**Methods:** We retrospectively analyzed data from pediatric patients with MDS, transplanted in our unit from June 1997 till February 2021. During this period, 38 patients, with a median age of 8.6 years (range 1.9-18 years) were treated with an allogeneic transplant. 20/38 were boys. Patients were classified according to EWOG-MDS criteria as follows: 23 patients were diagnosed with Refractory Cytopenia of Childhood, 12 patients with Primary MDS with excess blasts (MDS-EB) and 3 patients with secondary MDS following malignancy. 5 patients (13%) had an underlying genetic predisposition syndrome. As far as cytogenetics are concerned, 8/38 patients had monosomy 7 or deletion 7q and 3 patients had trisomy 8. Seven patients were subjected to a second transplantation, 6/7 due to relapse and 1/7 due to primary graft failure. One of these seven patients relapsed after 2^nd^ transplant and received a 3^rd^ graft. Therefore, total number of HSCTs performed was 46. Matched sibling donors (MSDs), matched unrelated donors (MUDs, HLA 10/10 or 9/10) and haploidentical donors (haplo) were used in 35%, 57% and 8% of cases respectively, whereas graft source was bone marrow, peripheral blood stem cells and cord blood in 67%, 29% and 4% cases respectively. Pre-transplantation conditioning was either busulfan(bu)-based [bu/cyclophosphamide(cy), bu/cy/melphalan(mel), bu/fludarabine(flu)/clofarabine(clo), bu/flu/thiotepa(thio)], or treosulfan(treo)-based (treo/flu, treo/flu/thio). Probabilities of Overall Survival (OS) and Disease-free-Survival (DFS) were estimated according to Kaplan-Meier method. Cumulative incidence of Transplantation Related Mortality (TRM), relapse, Graft versus Host Disease (GvHD) and complications, were calculated using the cumulative incidence estimator. Death due to any reason was considered as a competing event.

**Results**: Probability of OS was 47.3% and probability of DFS was 45.3%. Probability of OS differed significantly in patients transplanted after 2010 compared to those transplanted before 2010 – OS 71% vs 24%, p=0.002. Accordingly, probability of DFS was better in patients transplanted after 2010, 70.5% vs 23.5%, p=0.001. Cumulative incidence of post-transplantation relapse was 25.3%, with a median time of occurrence 203 days after graft infusion. Cumulative incidence of TRM at 100 days post HSCT was 10.5%. Cumulative incidence of TRM at 365 days post HSCT reached 26.3%. Main causes of death were relapse and infections, mainly fungal. Cumulative incidence of acute GvHD was estimated at 40% and that of aGvHD grade III at 17%. No patient developed aGvHD grade IV. Four patients exhibited chronic GvHD (8.5%): two patients localized skin cGvHD and two patients extensive cGvHD, involving skin, liver and bronchiolitis obliterans, that both died because of it.

**Conclusions:** HSCT represents a viable therapeutic solution for pediatric patients with MDS, with acceptable OS and DFS probabilities. Genetic testing, routinely performed in patients with MDS since 2015 determines the procedure of HSCT, with the aim of further improving therapeutic outcome.

## P10. Allogeneic Hematopoietic Stem Cell Transplantation in Pediatric Patients with Juvenile Myelomonocytic Leukemia (JMML) – The Greek Experience

### C. Oikonomopoulou, A. Paisiou, A. Komitopoulou, E.-D. Ioannidou, A. Kaisari, M. Kastamoulas, G. Stavroulaki, G. Vessalas, E. Goussetis, I.Peristeri

#### Stem Cell Transplant Unit, Aghia Sophia Children’s Hospital, Athens, Greece

##### **Correspondence:** C. Oikonomopoulou

**Introduction:** Juvenile Myelomonocytic Leukemia (JMML) is a pediatric leukemia sharing features of myelodysplastic and myeloproliferative neoplasms. Its clinical and hematological picture, as well as natural history and outcome, are remarkably diverse. Deregulation of the intracellular Ras signal transduction pathway is the predominant molecular feature of JMML and mutations of one of five genes (PTPN11, NRAS, KRAS, NF1, or CBL) are described in >90% of cases, further determining therapeutic decisions. Allogeneic hematopoietic stem cell transplantation (HSCT) is the only curative option for most patients.

**Aim:** Our aim was to retrospectively examine the outcome of children with JMML transplanted in our unit.

**Methods:** We retrospectively analyzed data from pediatric patients with JMML, transplanted in our unit from April 1998 till November 2020. During this period, 13 patients, with a median age of 2.2 years (range 6 months to 13.5 years) were treated with an allogeneic transplant. 10/13 were boys and 3/13 were girls. Three patients relapsed after HSCT and were given a second transplant from an alternative donor. Therefore, total number of HSCTs performed was 16. Matched sibling donors, matched related donors (HLA 9/10) and matched unrelated donors (HLA 10/10 or 9/10) were used in 7, 3 and 6 cases respectively, whereas graft source was bone marrow, peripheral blood stem cells and cord blood in 13, 2 and 1 cases respectively. Myeloablative conditioning was administered in all cases, more precisely Busulfan/Cyclophosphamide in 5 cases, Busulfan/ Cyclophosphamide/Melphalan in 7 cases and Treosulfan/Fludarabine/Melphalan or Thiotepa in 4 cases. Overall Survival (OS) and Disease free Survival (DFS) were estimated according to Kaplan-Meier method. Probabilities of Transplantation Related Mortality (TRM), rejection, Graft versus Host Disease (GvHD) and complications, were calculated using the cumulative incidence estimator. Death due to any reason was considered as a competing event.

**Results:** Probability of OS was 79% (95% CI 36.7%-94.7%) and probability of DFS was 63.5% (95% CI 28.9%-84.7%). Cumulative incidence of post-transplantation relapse was 33.7% (95% CI 9%-61%), with a median time of occurrence 158 days after graft infusion.

Cumulative incidence of TRM at 100 days post HSCT was 18.7% (95% CI 0%-35.8%) and was the same at 365 days post HSCT. Overall, two patients died, one due to transplantation toxicity at day +56 and the second due to septic shock at day +152 after his second transplantation attempt.

Cumulative incidence of acute GvHD was estimated at 57.7% (95% CI 18%-78%) and that of aGvHD grade III at 36% (95% CI 5%-59%). No patient developed aGvHD grade IV. Four patients exhibited chronic GvHD (31%): a case of bronchiolitis obliterans and three cases of mild cGvHD – one patient with liver cGvHD and two patients with localized skin cGvHD.

Incidence of hepatic veno-occlusive disease was 12.5%, incidence of CMV viremia 44% and prevalence of other infections was estimated at 31%.

**Conclusions:** HSCT using myeloablative non-TBI based conditioning for pediatric patients with JMML represents a curative therapy with high probabilities of overall survival and disease free survival. Genetic testing, routinely performed in newly diagnosed patients with JMML since 2015 is of great importance in general, but also regarding HSCT, as it determines conditioning regimens and additionally post transplantation surveillance, in order to minimize relapse.

## P11. Monocentric experience with pediatric patients with GATA2 deficiency

### G. Ovsyannikova, A. Pavlova, E. Deordieva, T. Konyukhova, M. Maschan, N. Smetanina

#### Dmitry Rogachev National Medical Research Center of Pediatric Hematology, Oncology and Immunology, Moscow, Russian Federation

**Keywords:** myeloid neoplasms with germline predisposition; leukemia*;* GATA2 deficiency.

**Introduction:** GATA2 deficiency is one of the most common predisposing conditions for MDS in young individuals. It is characterized by autosomal dominant inheritance with a high rate of *de novo* mutations. GATA2 syndrome exhibits incomplete penetrance and heterogeneity in clinical presentation with manifestation of myeloid neoplasia at any age. Here we describe the clinical phenotype and hematological presentation of 10 pediatric patients (pts) with GATA2 deficiency.

**Aim:** To analyze clinical and genetic data from pediatric pts diagnosed with GATA2 deficiency.

**Methods**: We performed a retrospective analysis of 10 pediatric pts 3.1 - 20.9 years of age, in whom a diagnosis of GATA2 deficiency had been made over a 7-year period (2013-2020). All patient samples had been obtained with informed consent, and the study had been approved by the local ethics committee.

Genetic testing was done by Sanger sequencing for single gene analysis or target next-generation sequencing (NGS) on the MiSeq/NextSeq (Illumina, USA) using a bone marrow failure custom gene panel.

**Results**: The 10 pts with GATA2 deficiency had been referred with a preliminary diagnosis of aplastic anemia or neutropenia. The median age at onset of cytopenia was 10.5 years (range 2.8-17.6). *GATA2* mutations in all pts were located in zink finger 2; there were 4 missense and 6 truncating mutations including 4 nonsense and 2 frameshift changes. Genetic analysis in non-hematological tissue demonstrated germline origin of the alteration in all six cases analyzed. All pts presented with a severely decreased absolute neutrophil count ranging between 0.32 – 1.10 G/L. In addition, all had monocytopenia with an absolute monocyte count between 0.01 - 0.18 G/L. None of the pts had a platelet count below 100 G/L (range 106 – 469). Median hemoglobin level was 10.8 g/dl (range 7.2 – 14.6), and one patient required red cell transfusions prior to diagnosis. In all pts, the blast percentage in the bone marrow was <5%, and in 8 out of the 10 cases the marrow was hypocellular at the first bone marrow evaluation. Five of the 10 pts had cytogenetic abnormalities (-7q/del, +8 trisomy) at the time of the first evaluation. Recurrent infections including one episode of a severe bacterial infection were observed in 8 pts. Eight out of the 10 pts had physical abnormalities like genitourinary tract abnormalities (n=3), lymphedema (n=2), cardiac malformation (n=2), biliary tract abnormality (n=1) and deafness (n=2). These data may indicate that genitourinary tract malformations are more common in the constitutional phenotype of GATA2 deficiency than previously appreciated. Four of the 10 pts received hematopoietic stem cell transplantation (HSCT). One patient was grafted from a matched unrelated donor (MUD), while the other 3 pts received a haploidentical graft since an MUD was not available. Two of the latter had primary graft failure requiring a second grafting procedure. At the time of writing, seven pts are alive, one patient died due to varicella zoster encephalitis, one patient died after the second HSCT due to infectious complications and another patient had disease progression and died due to infectious complications during intensive chemotherapy.

**Conclusion:** In this retrospective study we expand the clinical and genetic phenotype of GATA deficiency by outlining previously undescribed constitutional abnormalities and novel mutations. Thus, our work underlines the broad heterogeneity of the predisposition syndrome.

## P12. MDS progressing to CMML in a 8-year old girl with Fanconi anemia and mosaic trisomy of 8 chromosome

### K. Pawelec, E. Stanczak-Bujnicka

#### Department of Pediatric Hematology and Oncology Medical University od Warsaw, Warszawa, Poland

##### **Correspondence:** K. Pawelec

**Introduction:** Although Fanconi anemia (FA) is a rare mainly autosomal recessive genetic disease it is the common cause of inherited bone marrow failure revealing with pancytopenia and one of the most common genetic causes of hematologic malignancies such as myelodysplastic syndrome (MDS) and acute myelogenous leukemia (AML).

**Aim:** We presents the patient with both Fanconi anemia and mosaic trisomy 8, that developed MDS, and progressed to chronic myelomonocytic leukemia (CMML).

**Methods/Results:** 8 year old girl was admitted to Pediatric Oncology and Hematology Department for the diagnosis of pancytopenia lasting from about 4 months. One month earlier, the patient was hospitalized due to influenza B-related pneumonia and complications (respiratory failure and myocarditis). Patient’s coexisting conditions included: chromosomal aberration of 8 chromosome, congenital inappropriate exit of the left coronary artery from the pulmonary artery (Bland – White - Garland syndrome), rotated ectopic left kidney in the pelvis, myopia and strabismus. Blood tests on admission revealed pancytopenia, bone marrow examination with low cell count, and dysplastic forms. Test with mitomycin confirmed mosaicism of 8 chromosome trisomy and spontaneous chromosome breakages. Karyotype of parents confirmed the carrier of monoallelic deletion in FANCA gene. Patient was qualified to allogeneic hematopoietic stem cell transplantation (allo - HSCT) from unrelated donor because of MDS and FA was diagnosed 2 months later, the girl developed a fever without symptoms of infection, in control morphology test: anemia, WBC 31 x 10^3^/μl, PLT normal In blood smear – monocytosis 1,0x10^6^/μl, in bone marrow smear: highcellular bone marrow with blast cell 20%, Progression to CMML was diagnosed. The patient was classified to azacytidine therapy. After first cycle the patient experienced complications - fatigue, loss of appetite, fever progressed to septic shock and developed severe acute respiratory distress syndrome (ARDS). Treatment with azacytidine has been stopped. After 3 months the girl developed pneumonia again with negative bacterial and fungal cultures. Despite multi-drug therapy, she died 8 months after diagnosis of MDS / FA .

**Conclusions:** An earlier diagnosis of FA in a girl would be improve prognosis.

Consent to publish has been obtained.

## P13. Juvenile Myelomonocytic (JMML) leukemia in GREECE 2015-2021: the experience of the Greek study group for MDS/JMML/SAA, as a member of the EWOG-MDS/SAA working group

### L. Petrikkos^1^, N. Tourkantoni^2^, E. Mantadakis^3^, C.Kelaidi^1^, M. Servitzoglou^4^, E. Dikaia Ioannidou^5^, K. Tsitsikas^1^, K.Manola^6^, M. Kourti^7^, A. Tragiannidis^8^, E. Dana^9^, H. Tsipou^2^, N. Katzilakis^10^, K. Antoniadi^1^, M. Economou^11^, A.Makis^12^, M. Baka^4^, E. Papakonstantinou^7^, E. Chatzipantelis^8^, H.Kosmidis^9^, E.Stiakaki^10^, K.Stefanaki^13^, G.Paterakis^14^, I.Peristeri^5^, A. Kattamis^2^, S.Polychronopoulou^1^

#### ^1^Department of Paediatric Haematology-Oncology (Τ.Α.Ο.), «Aghia Sophia» Children’s Hospital, Athens, Greece; ^2^Division of Paediatric Hematology-Oncology, 1st Dpt of Pediatrics, National & Kapodistrian University Of Athens, «Aghia Sophia» Children’s Hospital, Athens, Greece; ^3^Department of Pediatrics, Democritus University of Thrace, University General Hospital of Alexandroupolis, Alexandroupolis, Greece; ^4^Oncology Department, “P&A Kyriakou” Children’s Hospital, Athens, Greece; ^5^Bone Marrow Transplantation Unit, «Aghia Sophia» Children’s Hospital, Athens, Greece; ^6^Laboratory of Health Physics, Radiobiology and Cytogenetics, National Center for Scientific Research «Demokritos» , Athens, Greece; ^7^Department of Paediatric Oncology, Hippokration General Hospital, Thessaloniki, Greece; ^8^Division of Paediatric & Adolescent Hematology-Oncology, 2nd Dpt of Paediatrics, Aristotle University of Thessaloniki, AHEPA General Hospital, Thessaloniki, Greece; ^9^Pediatric & Adolescent Oncology Clinic, “Mitera” Hospital, Athens, Greece; ^10^Department of Paediatric Hematology-Oncology, University Hospital of Heraklion, Heraklion. Crete, Greece; ^11^Division of Haematology, 1st Dpt of Paediatrics, Aristotle University of Thessaloniki, Hippokration General Hospital, , Thessaloniki, Greece; ^12^Department of Paediatrics, University of Ioannina, University Hospital, Ioannina, Greece; ^13^Department of Pathology, «Aghia Sophia» Children’s Hospital, Athens, Greece; ^14^Immunology Department, Flow Cytometry Laboratory, “G.Gennimatas” General Hospital, Athens, Greece

##### **Correspondence:** L. Petrikkos

**Introduction/Aim:** JMML is a rare clonal haematopoietic malignancy classified as a bridging disorder between myelodysplastic syndromes (MDS) and myeloproliferative diseases. Approximately 90% of patients carry either somatic or germline mutations in *GMCSF-receptor-RAS/MAPK* signaling pathway genes, which determine the therapeutic approach. For the better understanding, treatment and integration of new treatment options, the participation in national and European study groups is essential. We present the 6-year experience on JMML patients, of the Greek study group for MDS/JMML/SAA, as a member of the EWOG MDS/SAA European working group.

**Methods:** Six (6) JMML patients diagnosed according to international diagnostic criteria during the period 10/2015 – 7/2021 are presented. Appropriate detailed clinical, laboratory and cytogenetic investigations were performed. Diagnostic samples and clinical data were reviewed in national reference centers. The patients were treated in the paediatric hematology-oncology departments and the pediatric BMT-unit of Greece, and were consequently registered to EWOG-MDS/JMML.

**Results:** The median age of the 6 patients at diagnosis were 18,6 months. Four out of six were males. At diagnosis: median WBC 26725/μL, median AMC 6217/μL, median Hb 9.75gr/dl, median PLTs 39500/μL and median HbF 13.7%. All patients presented with myeloid precursors in blood. Median percentage of blasts in blood was 4% (range 1-7%) and in bone marrow 9.3% (range 3-17%). Spontaneous growth or granulocyte-macrophage colony-stimulating factor (GM-CSF) hypersensitivity in colony assay was detected in all patients. All patients had splenomegaly, with respiratory symptoms 4/6 patients, rash 2/6 patients, dysmorphic features or/and genetic stigmata 4/6 (one with CBL syndrome’s features, and one with Costello-like syndrome’s features). Mutations in genes of *GMCSF-receptor-RAS/MAPK* signaling pathway were detected in all patients. Cytogenetics and molecular findings and therapeutic approach is presented in the Table. The patient n.4 relapsed 12.3 months after SCT, received azacitidine+ventoclax followed by a second SCT. All patients are alive (OS 100%) in remission without transfusion needs under close follow up (median time from diagnosis 44months)

**Conclusions:** JMML is characterized by special clinical, laboratory and molecular features. The pathogenesis lies in the deregulation of the RAS signaling pathway. The prognosis of JMML is strongly associated with genetic mutations. HSCT is the treatment of choice for most patients, but for some patients a watch-and-wait strategy is recommended. Current and further studies may identify the role of demethylatitng agents (eg azacitidin) and targeted therapy for JMML patients. In this direction and due to the rarity of the disease, international cooperation is fundamental.

**Table 1 (abstract P13).** See text for description

	Karyotype	Mutated gene	Therapeutic approach
Patient-1	45,XY,-7[8]/46,XY[2]	*KRAS* (somatic)	Cytoreduction (6-MP/AraC) + SCT
Patient-2	46,ΧΧ[25]	*NRAS* (germline)	6-MP (for 3 years) then watchful waiting
Patient-3	47,XYY,1qh+[25]	*CBL* (germline)	Watchful waiting
Patient-4	46,ΧΥ[25]	*KRAS* (somatic)	Azacitidine+SCT, Azacitidine+venetoclax+2nd SCT
Patient-5	46,ΧΥ[25]	*RRAS2* (germline)	Watchful waiting
Patient-6	46,ΧΧ[19]	*PTPN11* (somatic)	Azacitidine+SCT

## P14. Identification of novel therapeutic approaches in a xenograft model of juvenile myelomonocytic leukemia (JMML)

### J. Rajak^1^, N. Koleci^1^, Y. Wu^1^, T. Watrin^2^, S. Bhatia^2^, A. Borkhardt^2^, C. Flotho^1^, C.M. Niemeyer^1^, M. Erlacher^1^

#### ^1^University Medical Center Freiburg, Center for Pediatrics Department of Pediatric Hematology and Oncology, Freiburg, Germany; ^2^Department of Pediatric Oncology, Hematology and Clinical Immunology, Medical Faculty, Heinrich Heine University Düsseldorf, Düsseldorf, Germany

**Introduction:** Juvenile myelomonocytic leukemia (JMML) is a highly aggressive myeloproliferative disorder of early childhood and the only curative treatment is hematopoietic stem cell transplantation (HSCT). However, the main cause of treatment failure is relapse in up to 30% of patients urging the strong need for novel therapies. In JMML, malignant transformation is driven by constitutive activation of the RAS signaling pathway providing the rationale for its therapeutic inhibition. By using primary JMML cells and our patient-derived xenograft (PDX) model that closely mimics human disease, we are testing different novel targeted therapies to identify novel treatment strategies able to cure JMML.

**Methods/Results:** We performed drug screening assays on primary JMML samples with a library of anticancer compounds including both clinically approved and investigational drugs. Drug sensitivity results were heterogenous, but consistent sensitivity was observed to heat shock protein 90 inhibitors, histone deacetylase inhibitors, FLT3 tyrosine kinase inhibitors and polo-like kinase inhibitors. In addition, several other interesting compounds targeting (in)directly RAS signaling and tested in adult leukemias but not included in the drug screening assay, were tested *in vitro* for their cytotoxicity towards JMML cells. Our selection included Idelalisib (PI3K inhibitor), LY3009 (pan-RAF inhibitor), Pevonedistat (NEDD8 activation enzyme inhibitor) and Ruxolitinib (JAK1/2 inhibitor). JMML cells showed high sensitivity to Idelalisib and Pevonedistat. In contrast, LY3009 and Ruxolitinib did not influence or had little effects on cells, respectively. Our *in vitro* data on Pevonedistat as promising novel candidate will be further corroborated in engrafted mouse model of JMML. In vivo studies will be conducted in established JMML xenograft Rag2-/-γc-/- mouse model that nicely imitates disease features. Newborn mice will be irradiated with 2.5 Gy and intrahepaticaly injected with 1x10^6^ of JMML mononuclear cells. Eight weeks after transplantation mice will be subjected to Pevonedistat. Treated mice will be thoroughly analyzed by flow cytometry and histology. By serial transplantation assays, the effects of the drug specifically on leukemia-initiating cells will be tested. In a different line of investigation, the immunosuppressive effects of JMML cells will be analyzed, and checkpoint inhibitors (i.e. PD-L1 inhibitor, anti-CD47) will be used in vitro assays and, whenever possible, in our PDX mice.

**Conclusions:** It is our primary goal to deplete JMML-initiating cells to prevent relapse after HSCT. Eventually, we plan to transfer our preclinical observations to phase I/II clinical trials and in that way improve care of JMML patients. In addition, our studies will contribute to a better understanding of pathogenetic mechanisms of JMML.

## P15. Bone marrow failure and myelodysplastic syndromes in a level III pediatric hospital: a 10 year retrospective study

### J. Ribeiro^1^**,** M. Matias^1^, M. Coucelo^2^, J.-P. Gonçalves^3^, M. Oliveira^1^, S. Batalha^1^, R. Maia^1^, P. Kjöllerström^1^

#### ^1^Unidade de Hematologia Pediátrica, Hospital Dona Estefânia, Centro Hospitalar Universitário de Lisboa Central, Lisboa, Portugal; ^2^Laboratório de Hematologia Molecular do Serviço de Hematologia Clínica, Centro Hospitalar e Universitário de Coimbra, Coimbra, Portugal; ^3^Serviço de Pediatria, Instituto Português de Oncologia Francisco Gentil, Lisboa, Portugal

**Introduction:** Bone marrow failure syndromes (BMFS) and myelodysplastic syndromes (MDS) are rare disorders that condition the normal differentiation of hematopoietic stem cells. The first group is characterized by inherited or acquired impaired production of blood cells; while the latter presents mainly with morphologic dysplasia of bone marrow cells. BMFS often present in childhood, whereas MDS are more common in adults; in children they are frequently associated with inherited forms of BMFS or predisposition syndromes. As some of these morphological changes overlap, definitive diagnosis may be hard to obtain and should be carried out by experienced teams in order to better predict risk of leukemic transformation and select the best treatment and follow up strategy.

**Aim:** To review all BMFS and/or MDS Pediatric cases evaluated in a Pediatric Hematology (non-oncology) Unit of a level III Pediatric Hospital.

**Methods:** Descriptive analytical retrospective study, covering a 10 year period (2010-2020). Inclusion criteria were BMFS and/or MDS diagnosis, with age of onset and follow-up period until 18 years old. No patient was excluded. The variables analyzed were demographic, clinical, laboratory and therapeutic. Statistical analysis was performed using SPSS 22®.

**Results:**We reviewed a total of 38 cases, 55% male, with a median age of 6 years (IQR 11). 74% (28) were classified as BMFS and 26% (10) MDS. Regarding classification of BMFS (28): half of the patients (14) had congenital disease with predisposition to myelodysplasia; 57% (8) were male, median age of 3 years (IQR 4); 71% (10) had dysmorphisms. 36% had pancytopenia, 28% bicytopenia and in 36% only one affected lineage. We identified 5 patients with Fanconi anemia, 4 Blackfan Diamond Syndrome, 1 congenital amegakaryocytic thrombocytopenia, 1 dyskeratosis congenita, 1 Shwachman- Diamond syndrome, 1 DNA ligase IV syndrome and 1 radioulnar synostosis syndrome. 50% received hematopoietic cell transplantation (HCT). In the other 50% (14) a predisposing genetical condition was not identified; 71% (10) were male, median age of 10 years (IQR 11), 7% (1) with phenotypical abnormalities, 86% had pancytopenia and 14% bicytopenia; 50% received HCT and 21% immunosuppressive treatment. Regarding MDS patients (10): 50% were male, with a median age of 6,5 years (IQR 11), 20% had dysmorphic features. 50% presented with pancytopenia, 20% bicytopenia and 30% one lineage cytopenia. Three of them had a genetic diagnosis: 2 *SAMD9* and 1 *RUNX1*. Four of them had leukemic transformation; all had bone marrow cytogenetic abnormalities. 40% underwent HCT.

**Conclusions:** Our case series reflects the expected age and pathology distribution for BMFS and MDS in childhood. Adequate clinical assessment, morphology, cytogenetics and molecular biology are key in identifying a growing number of previously underdiagnosed pathologies, with different risk and management strategies. A multidisciplinary approach has been fundamental to assure a faster and more accurate diagnosis with increasing better outcomes.

## P16. A new analysis pipeline to investigate the structural variants derived from RNA-seq data of AML patients

### I. Şahin^1,2^, Ö. Özdemir ^1,3^, Ö. Hatırnaz Ng^1,4,5^, U. Özbek^1,3,4^

#### ^1^Acıbadem University Rare Diseases and Orphan Drugs Application and Research Center (ACURARE); ^2^Acıbadem Mehmet Ali Aydınlar University, Institute of Health Sciences, Department of Medical Biotechnology; ^3^Acıbadem Mehmet Ali Aydınlar University, Institute of Health Sciences, Genome Studies Program; ^4^Acıbadem Mehmet Ali Aydınlar University, Faculty of Medicine, Department of Medical Genetics; ^5^Acıbadem Mehmet Ali Aydınlar University, Faculty of Medicine, Department of Medical Biology

**Introduction:** AML is a heterogeneous hematologic malignancy with a complex pathogenesis involving a wide variety of molecular modifications. Molecular genetic alterations underlie the pathogenesis of AML; therefore, understanding these processes is vital for developing disease- specific treatments. AML contains various abnormalities such as SNVs, large insertions and deletions, fusion genes, and structural chromosomal aberrations. In addition to genetic variants, gene expression levels play a role in the prognosis of the disease. All these features cause heterogeneity not only in terms of the clinical course of the disease, but also in treatment response and the prognosis of the disease. Accurate classification of AML is essential for eligible treatment and elucidating all of the heterogeneity requires the utilization of different techniques. Even though comprehensive assessment of AML risk classification and diagnosis is critical, in practice, it is complex, costly, and mostly delayed due to the complexity of the disease and the workload. The long period of molecular diagnosis is an obstacle to considering the mutational profile of AML and therefore reaching the appropriate treatment for patients. In addition, although chromosomal rearrangements and copy number variations remain the basis for the diagnosis of AML, the information obtained as a result of molecular genetic analysis is consequential for 50% of adult patients presenting with a normal karyotype.

**Aim:**The purpose of the study is to develop an analysis and reporting pipeline for ultra-rapid and accurate AML molecular classification with a single experimental process. With the whole transcriptome sequencing, it will be possible to evaluate the structural variants, expression profiles, and translocations in one AML-variome panel.

**Methods:** Paired-end RNA sequencing data from AML patients as well as cell lines were retrieved from Gene Expression Omnibus. Read pairs were aligned to the human reference genome (GRCh38) using STAR (2.7.9a). Subsequently, aligned .bam files were addressed to the SQUID algorithm for detection of the AML-related structural variants. The output file was filtered for the gene fusions according to the WHO 2016 classification and ELN 2017 risk stratification. To reduce the false positive/negative results and confirm the detected translocations, novel reference assemblies were created specific to the AML structural variations via BamSurgeon. Sequences that include structural variants were realigned to the novel assembly. After the IGV validation, detected structural variants were plotted using the Samplot tool. The benchmarking of the pipeline was also performed with the simulated data that were created from GEO datasets and specific to the AML variants.

**Results:** We have developed an analysis pipeline that can successfully detect reported SVs with known prognostic relevance for AML. The pipeline has been tested in various GEO datasets including different cases and cell lines with main clinically relevant genetic alterations as well as simulated data. Filtering criteria revealed accurate results for known translocations including *PML-RARA*, *MYH11-CBFB*, *RUNX1-RUNX1T1*, and *MLLT3-KMT2A.*

**Conclusions:** This study aimed to develop an analysis and reporting pipeline for rapid and accurate AML molecular classification with a single experimental process via whole transcriptome sequencing. The first phase of the new pipeline which was the detection of the SVs was successfully completed.

## P17. PTPN11 mutations in juvenile myelomonocytic leukemia and Noonan syndrome with transient myeloproliferative disorder – diagnostic challenges and pitfalls

### S.Salou^1^, M. Kaiser^1^, D. Lebrecht^1^, D. Yilmaz Karapinar^2^, M. Lauten^3^, S. Holzhauer^4^, A. Yoshimi-Nöllke^1^, M. Erlacher^1,5,6^, C.M. Niemeyer^1,5,6^, C. Flotho^1,5,6^

#### ^1^Division of Pediatric Hematology and Oncology, Department of Pediatrics and Adolescent Medicine, Medical Center, Faculty of Medicine, University of Freiburg, Freiburg, Germany; ^2^Department of Pediatric Hematology and Oncology, Ege University School of Medicine, İzmir, Turkey; ^3^Department of Pediatric and Adolescent Medicine, Pediatric Hematology and Oncology, University Hospital Schleswig-Holstein, Lübeck, Germany; ^4^Charité, University Medicine, Pediatric Hematology and Oncology, Berlin, Germany; ^5^German Cancer Consortium (DKTK), partner site Freiburg, Germany; ^6^German Cancer Research Center (DKFZ), Heidelberg, Germany

**Introduction:** Somatic mutations in the *PTPN11* gene are the most frequent genetic aberration in juvenile myelomonocytic leukemia (JMML) whereas germline mutations in the same gene are found in Noonan syndrome (NS)/transient myeloproliferative disorder (MPD). Since the natural course and treatment regimen of these two conditions differ significantly, the early and reliable distinction of somatic and germline mutation is crucial.

**Methods/Results:** In a retrospective ten-year analysis of newly diagnosed JMML and NS/MPD cases enrolled in the EWOG-MDS 2006 study we reviewed all patients with *PTPN11* mutations. Among 278 patients, 243 were tested negative in non-hematopoietic tissue and thus diagnosed as JMML. Thirty-five patients had evidence of a germline mutation and were registered as NS/MPD. There was a broad overlap of mutated amino acid residues between JMML and NS/MPD, indicating that genotype-phenotype correlation is not possible in these patients. Analysis of cells from buccal swabs often gave positive results that could not be confirmed in other non-hematopoietic materials such as hair follicles or skin fibroblasts. We assume that this was probably due to infiltrating leukocytes and therefore recommend caution when evaluating buccal swabs. They should only be considered useful if they are clearly negative. Therefore, hair follicles seemed to be the preferable material. However, we encountered three cases in the last three years where the analysis of hair follicles was positive for the same *PTPN11* mutation as found in blood, although the mutation was later not confirmed in fibroblast cultures from skin biopsies nor in buccal swabs. In one case, monosomy 7 present in the hematopoietic system could also be detected in hair follicle DNA, suggesting contamination with leukocytes. While these children partly presented with clinical features of NS, none of them showed the most suggestive features like pulmonary valve stenosis or characteristic facial dysmorphism.

**Conclusions:** In summary, we suggest that NS/MPD may not be reliably diagnosed or excluded by analyzing buccal swabs or hair follicles alone. Instead, we propose the diagnostic analysis in at least two independent non-hematopoietic tissue samples, or in cultured fibroblasts as the gold standard. Special awareness is required in patients with a positive result in non-hematopoietic tissue but absence of suggestive syndromic features.

